# Taxonomic and functional partitioning of Chloroflexota populations under ferruginous conditions at and below the sediment-water interface

**DOI:** 10.1093/femsec/fiae140

**Published:** 2024-10-09

**Authors:** Aurèle Vuillemin, Fatima Ruiz-Blas, Sizhong Yang, Alexander Bartholomäus, Cynthia Henny, Jens Kallmeyer

**Affiliations:** GFZ German Research Centre for Geosciences, Section Geomicrobiology, Telegrafenberg, 14473 Potsdam, Germany; GFZ German Research Centre for Geosciences, Section Geomicrobiology, Telegrafenberg, 14473 Potsdam, Germany; GFZ German Research Centre for Geosciences, Section Geomicrobiology, Telegrafenberg, 14473 Potsdam, Germany; GFZ German Research Centre for Geosciences, Section Geomicrobiology, Telegrafenberg, 14473 Potsdam, Germany; Research Center for Limnology and Water Resources, National Research and Innovation Agency (BRIN), Cibinong, 16911 Jawa Barat, Indonesia; GFZ German Research Centre for Geosciences, Section Geomicrobiology, Telegrafenberg, 14473 Potsdam, Germany

**Keywords:** Chloroflexota, Anaerolineae, Dehalococcoidia, ferruginous conditions, sulfate reduction, fermentation, acetogenesis, Wood–Ljungdahl pathway

## Abstract

The adaptation of the phylum Chloroflexota to various geochemical conditions is thought to have originated in primitive microbial ecosystems, involving hydrogenotrophic energy conservation under ferruginous anoxia. Oligotrophic deep waters displaying anoxic ferruginous conditions, such as those of Lake Towuti, and their sediments may thus constitute a preferential ecological niche for investigating metabolic versatility in modern Chloroflexota. Combining pore water geochemistry, cell counts, sulfate reduction rates, and 16S rRNA genes with in-depth analysis of metagenome-assembled genomes, we show that Chloroflexota benefit from cross-feeding on metabolites derived from canonical respiration chains and fermentation. Detailing their genetic contents, we provide molecular evidence that Anaerolineae have metabolic potential to use unconventional electron acceptors, different cytochromes, and multiple redox metalloproteins to cope with oxygen fluctuations, and thereby effectively colonizing the ferruginous sediment-water interface. In sediments, Dehalococcoidia evolved to be acetogens, scavenging fatty acids, haloacids, and aromatic acids, apparently bypassing specific steps in carbon assimilation pathways to perform energy-conserving secondary fermentations combined with CO_2_ fixation via the Wood–Ljungdahl pathway. Our study highlights the partitioning of Chloroflexota populations according to alternative electron acceptors and donors available at the sediment-water interface and below. Chloroflexota would have developed analogous primeval features due to oxygen fluctuations in ancient ferruginous ecosystems.

## Teaser

Chloroflexota populations are partitioned according to alternative electron acceptors (Anaerolineae) and donors (Dehalococcoidia) among respiratory and fermentative metabolites.

## Introduction

The phylum Chloroflexota is environmentally widespread and abundant in both aquatic and terrestrial habitats, whether photic or dark (Mehrshad et al. 2018b, Wiegand et al. 2023). Ecological niches in which they thrive encompass hot springs (Reysenbach and Cady 2001), sinkholes (Thomas et al. 2024), deep aquifers (Hug et al. 2013), contaminated soils (Sheik et al. 2012), Arctic and Antarctic regolith (Wilhelm et al. 2011, Williams et al. 2024), marine and lacustrine shallow to deep sediments (Blazejak and Schippers 2010, Vuillemin et al. 2018a, 2020), as well as hadal oceanic waters and clays (Liu et al. 2022, Wu et al. 2023). Despite their ubiquity, the limited cultivability of many groups of Chloroflexota hinders the study of their biology and evolution (Solden et al. 2016), and new metagenome-assembled genomes (MAGs) give a clearer picture of their versatile metabolisms (Petriglieri et al. 2023, Wiegand et al. 2023). Nevertheless, the reasons for the broad ecological distribution and diversity of (an)aerobic (photo)heterotrophs within the phylum Chloroflexota remain rather elusive (Kaster et al. 2014, Mehrshad et al. 2018b).

The classes Anaerolineae and Dehalococcoidia are the two main lineages found in anoxic sediments (Fincker et al. 2020). Some of the main metabolic processes that they can drive are anaerobic organohalide respiration via reductive dehalogenation (Futagami et al. 2009, Yang et al. 2020a), fermentation of carbohydrates (Yamada et al. 2006, 2007) and proteinaceous necromass (Vuillemin et al. 2020, Liu et al. 2022), degradation of complex polymeric organic compounds (Speirs et al. 2019), as well as substrate-level phosphorylation (SLP) of sugars and volatile fatty acids (VFAs) combined with (homo)acetogenesis (Hug et al. 2013, Sewell et al. 2017). In addition, the presence of genes encoding dissimilatory sulfite reductase (*dsr*) and reversible adenylylsulfate reductase (*apr*), along with genetic adaptation against environmental oxidative and osmotic stress, further imply sulfur cycling and respiration (Wasmund et al. 2016, Mehrshad et al. 2018a). Finally, they display enhanced resistance to several metals and metalloids (Sheik et al. 2012, Thomas et al. 2024), suggesting a metabolic capability for dissimilatory reduction of these compounds (Hori et al. 2015, Zhang et al. 2023). The adaptation of modern clades to such variety of geochemical contexts (Yang et al. 2020b, Bovio-Winkler et al. 2023) is thought to derive from ancestral genomic capabilities inherent to the gradual oxygenation of early ecosystems (West-Roberts et al. 2021), as well as energy conservation under ferruginous anoxia in much less productive non-photosynthetic microbial ecosystems (Knoll et al. 2016, Sauterey et al. 2020).

Here, we investigate environmental clades of Chloroflexota in anoxic ferruginous (iron-rich, sulfate-poor) sediments (Vuillemin et al. 2023) deposited under conditions considered to be analogous to those of Earth’s early oceans during the Proterozoic (Poulton and Canfield 2011). We report on their population abundance, taxonomic diversity, and metabolic functions, and further assess the specificity of selective genetic traits based on their MAGs with regard to organic matter (OM) mineralization under limited sulfate and high dissolved ferrous iron concentrations (Friese et al. 2021). Knowledge of microbially driven biogeochemical cycles in modern ferruginous ecosystems (Vuillemin et al. 2018b) can contribute to understanding the reasons for the wide environmental distribution of modern clades of Chloroflexota, and trace the emergence of their polyvalent mixotrophic metabolisms and cryptic respiration capabilities in Earth’s early oceans (Garber et al. 2021).

## Materials and methods

### Lake Towuti as ferruginous case study

**Figure 1.**
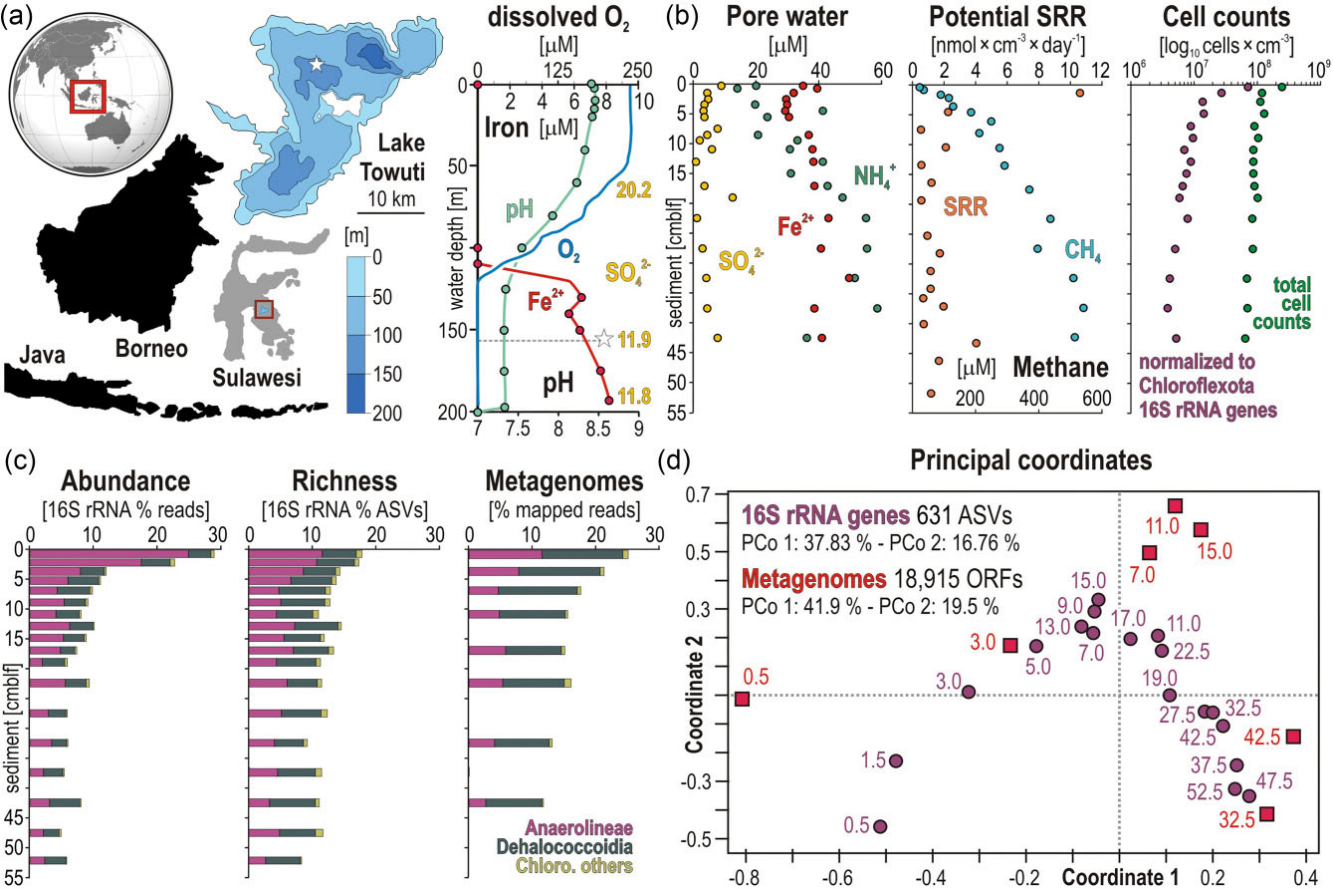
Site description of Lake Towuti, its water column and pore water geochemistry, characterization of microbial populations, alpha and beta diversities. (a) World map displaying the location of Sulawesi Island and Malili Lake System (red squares), with bathymetric map of Lake Towuti and position of the core site (white star) corresponding to 156 m water depth, and water column profiles for pH, O_2_, Fe^2+^, and SO_4_^2−^ concentrations. (b) SO_4_^2−^, Fe^2+^, and NH_4_^+^ pore water profiles; sulfate reduction rates (SRR) and methane (CH_4_) concentrations; total cell counts and total cell counts normalized to the relative abundance of 16S rRNA genes of Chloroflexota; (c) relative abundance (% reads) and richness (% ASVs) of 16S rRNA genes, and metagenomic reads taxonomically assigned to Chloroflexota; and (d) Principal Coordinates Analysis (PCoA) based on 631 ASVs (16S rRNA genes) and 18 915 ORFs (metagenomes) assigned to Chloroflexota.

Tropical Lake Towuti (2.5°S, 121.3°E) is the largest of the Malili Lakes, a chain of five interconnected tectonic lakes hosted in ophiolitic and ultramafic rocks on Sulawesi Island, Indonesia (Fig. 1a). Erosion of lateritic soils in the catchment supplies these lakes with little sulfate but appreciable amounts of detrital iron (oxyhydr)oxides (Morlock et al. 2019) that scavenge phosphorus in the water column (Zegeye et al. 2012). Modern Lake Towuti has a water depth of 200 m and is currently stratified with anoxic ferruginous bottom waters (Fig. 1a), wherein a substantial part of the sinking OM is remineralized through microbial reduction of ferrihydrite (Bauer et al. 2020). Previous geomicrobiological investigations showed that despite extremely low sulfate concentrations (<20 µM), biogeochemical processes related to sulfur, iron, and methane co-occur in Lake Towuti’s sediments (Vuillemin et al. 2016, 2018b, Friese et al. 2021).

### Field sampling, sediment aliquoting, and geochemical analyses

Field sampling took place in October 2013, November 2014, and June 2015, allowing the study of geochemical conditions in the water column and collection of sediment gravity cores. The water column’s oxygen concentration profile was measured using a submersible Sea-Bird SBE-19 conductivity–temperature–depth probe (Sea-Bird Electronics, Bellevue, WA, USA). The Fe^2+^ concentration profile (Fig. 1a) was obtained from water samples collected using 5 L Niskin bottles (General Oceanics, Miami, FL, USA). The upper 55 sediments below lake floor (cmblf) were retrieved via gravity coring. In the field, gravity cores were transferred into an anaerobic glove bag and sediments subsampled for methane concentration (2 cm^3^), pore water geochemistry (50 cm^3^), total cell count (2 cm^3^), sulfate reduction rates (3 × 5 cm^3^), and DNA extraction. Samples for methane were taken with a cutoff syringe and transferred into crimp vials filled with saturated NaCl solution without headspace. Sample resolution was 1 cm for the upper 5 cmblf, 2 cm for 5–20 cmblf, and 5 cm below. For DNA samples, a separate gravity core was sampled by placing bulk sediment into heat-sealed gas-tight aluminum foil bags flushed with nitrogen and stored at room temperature (25°C), close to that of lake bottom waters (28°C), until analysis. All sampling procedures and geochemical data were previously published (Vuillemin et al. 2016, Bauer et al. 2020, Friese et al. 2021).

Pore water was extracted in the glove bag from 50 ml fresh sediments, using Rhizon Pore Water Samplers (Rhizosphere research products, Wageningen, Netherlands), sterile-filtered, transferred into plastic vials with screw caps (Cryo Vials, Carl Roth, Karlsruhe, Germany), and stored at 4°C until analysis. Pore water SO_4_^2−^ and NH_4_^+^ concentrations were analyzed in triplicates, using non-suppressed (cations) and suppressed (anions) ion chromatography (Vuillemin et al. 2016). Detection and quantification limits of the method are 2.0 and 8.4 µM for SO_4_^2−^, and 11.3 and 67.6 µM for NH_4_^+^. Dissolved Fe^2+^ concentrations were measured in the field, transferring 1 ml of pore water aliquots to 1.5 ml Rotilabo single-use cells (Carl Roth, Karlsruhe, Germany). Dissolved Fe^2+^ was stabilized with 100 µL of Ferrozine Iron Reagent (Sigma-Aldrich Chemie, Taufkirchen, Germany) and the absorbance of the colored solution measured at 562 nm (Viollier et al. 2000) with a DR 3900 spectrophotometer (Hach, Düsseldorf, Germany).

For methane concentrations, we introduced 3 ml of helium as headspace 24 h prior to analysis and injected 200 µL of headspace into a Thermo Finnigan Trace gas chromatograph (Thermo Fisher Scientific), as published (Friese et al. 2021).

### Total cell counts, sulfate reduction rates

For total cell counts, 2 cm^3^ of sediment were fixed in 8 ml formalin solution (final concentration 2%). We mixed 50 µL of this slurry with 50 µL detergent mix (36.8 g L^−1^ Na_2_ EDTA × 2 H_2_O, 22.3 g L^−1^ Na-pyrophosphate × 10 H_2_O, 5 ml TWEEN 80), 50 µL methanol, and 350 µL ultrapure H_2_O, and processed in triplicates (Kallmeyer et al. 2008). To dissolve fine mineral particles, 50 µL of this solution were mixed with 5 µL of 1% hydrofluoric acid (Morono et al. 2009). The solution was filtered onto black 0.2 µm polycarbonate Cyclopore membrane filters (Whatman International Ltd., Maidstone, UK) and cells stained with SYBR Green I (Molecular Probes, Eugene, OR, USA), and counted by epifluorescence microscopy (Leica DM2000 microscope, Wetzlar, Germany), as previously published (Vuillemin et al. 2016).

Sulfate reduction rates (SRR) were determined by incubation of mechanically undisturbed sediment with radioactive ^35^SO_4_^2−^ tracer, using sterile glass mini-cores (Fossing et al. 2000, Friese et al. 2021). The microbially reduced inorganic sulfur species were extracted using cold chromium distillation (Kallmeyer et al. 2004), radioactivity in the extracts was quantified with a Tri Carb 2500 TR liquid scintillation counter (Packard Instruments, Fallbrook, CA, USA) using Ultima Gold Scintillation Cocktail (Perkin Elmer, Waltham, MA, USA).

### DNA extraction, 16S rRNA gene, and metagenome processing

Total DNA was extracted from 1 g of sediment using the GeneMATRIX Environmental DNA and RNA Purification Kit (EURx^®^, Gdánsk, Poland), following the manufacturer’s instructions. PCR amplification was performed as previously published (Vuillemin et al. 2017), using unique combinations of the barcoded universal primer pair 515F (5′-GTG TGY CAG CMG CCG CGG TAA-3′) with 806R (5′-CCG GAC TAC NVG GGT WTC TAA T-3′). The PCR products were cleaned using the HighPrep PCR Clean-up system (MAGBIO, Lausanne, Switzerland) and sent to Novogene (www.novogene.com) for sequencing of 16S rRNA gene partial amplicons (2 × 250 bps) on an Illumina NovaSeq platform. Read demultiplexing was performed using Cutadapt v. 3.5 (Martin 2011) with the following parameters: −e 0.2 − q1515 − m 150—discard-untrimmed. The ASVs were generated using trimmed reads and the DADA2 package v. 1.20 (Callahan et al. 2016) with R v. 4.1, applying the pooled approach with the following parameters: truncLen = c(220 180), maxN = 0, rm.phix = TRUE, minLen = 160. Taxonomic assignment was done against the SILVA 16S rRNA SSU database release 138 (Quast et al. 2013), using DADA2. ASVs representing chloroplasts, mitochondria, and singletons were removed. Sequencing of 16S rRNA gene libraries yielded identical results as those performed on biological replicates in 2015 (Vuillemin et al. 2017, 2018b). The 16S rRNA gene amplicons assigned to Chloroflexota were selected for downstream analysis (Fig. 1c).

Metagenomes were generated from DNA extracts obtained from 8 sediment samples (at 0–1, 2–4, 6–8, 10–12, 14–16, 20–25, 30–35, and 40–45 cmblf), using the Nextera XT DNA Library Preparation kit (Illumina, San Diego, CA, USA). Sequencing was performed on a NovaSeq 6000 Illumina platform at CeGaT GmbH (Tübingen, Germany), aiming for 50 million read pairs (2 × 150 bps) for each sample. Demultiplexing of the sequenced metagenomic libraries was performed with bcl2fastq v. 2.20. Adapters were trimmed with Skewer v. 0.2.2 (Jiang et al. 2014) and the FASTQ files quality-checked using FastQC v. 0.11.5. To obtain a taxonomic overview of the metagenomes (Fig. 1c), the quality-controlled reads were mapped to the SILVA 16S rRNA SSU database release 138 (Quast et al. 2013), using Bowtie2 (Langmead and Salzberg 2012). Metagenomic reads were further processed for quality control, *de novo* assembly of contigs, gene annotation, binning into MAGs, and taxonomic annotation, using ATLAS v. 2.1.0 (Kieser et al. 2020). Metagenomes were initially processed separately, then combined to improve the completeness of MAGs. The integrated ATLAS pipeline uses quality-controlled reads from BBTools (Bushnell 2014) to successively execute metaSPAdes v. 3.11.1 (Nurk et al. 2017) for contig assembly, Prodigal v. 2.6.3 (Hyatt et al. 2010) for Open Reading Frames (ORFs) extraction with eggNOG-mapper v. 2.1. (Cantalapiedra et al. 2021) for functional annotation, MetaBAT v. 2.1.5 (Kang et al. 2019) with MaxBin v. 2.2.7 (Wu et al. 2016) and DAS Tool v. 1.1.6 (Sieber et al. 2018) for binning into MAGs, and CheckM v.1.1.10 (Parks et al. 2015) to determine the level of MAG completeness and contamination. Taxonomic assignments of the MAGs were performed against the GTDB Genome Taxonomy Database v. 2.1.1 (Parks et al. 2022).

Predicted ORFs were extracted from both contigs assembled from separate metagenomes and 16 MAGs that were taxonomically assigned to Chloroflexota. Functional marker genes were identified by applying a bioinformatics pipeline that integrates functional annotations with best hit taxonomy by performing BLASTp searches of all extracted ORFs against a large aggregated genome database (Orsi et al. 2022), using the DIAMOND protein aligner v. 0.9.24 (Buchfink et al. 2015). The database contains 37.8 million predicted proteins compiled from the SEED (www.theseed.org) and NCBI RefSeq databases updated with high-quality MAGs and single-cell assembled genomes (SAGs) from the NCBI protein database.

### Phylogenetic analysis of 16S rRNA and functional genes

**Figure 2.**
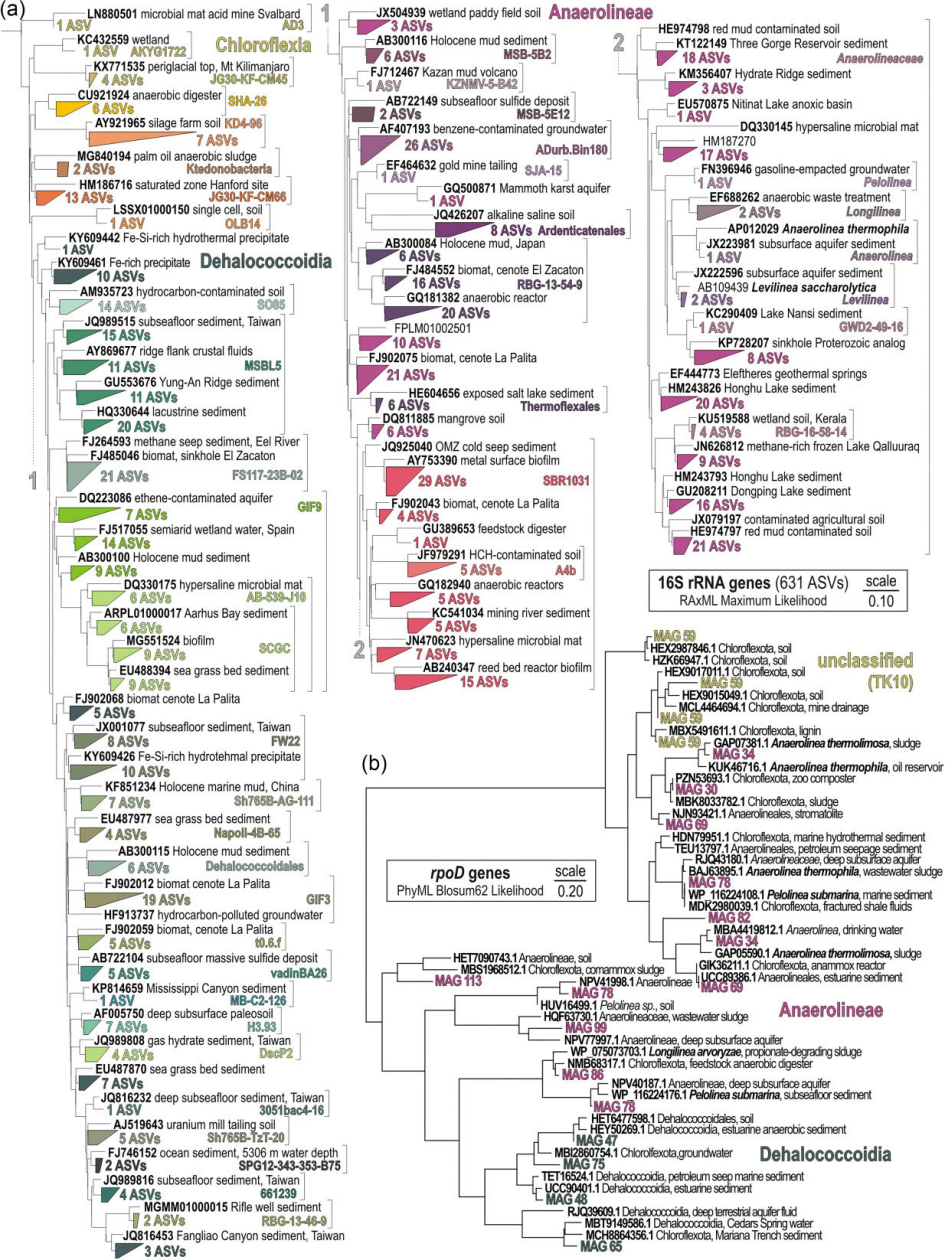
Phylogenetic analyses of 16S rRNA and *rpoD* protein-encoding genes. (a) RaxML maximum likelihood phylogenetic tree of partial 16S rRNA genes (V4 hypervariable region) taxonomically assigned to Chloroflexota based on 100 replicates; and (b) PhyML Blosum62 phylogenetic tree of ORFs for conserved regions (167 amino acids) encoding the RNA polymerase sigma 70 factor (*RpoD*) assigned to Chloroflexota based on 100 replicates. Boldface types signify cultivated species and sequence accession numbers to the SILVA and NCBI databases.

Two Principal Coordinates Analyses (PCoA) were performed with the Bray–Curtis similarity index, using Past v. 4.03 (Hammer et al. 2001). The first calculation was based on 631 ASVs generated from 16S rRNA gene amplicons, the second one on 18 915 ORFs extracted from contigs assigned to Chloroflexota (Fig. 1d). The 16S rRNA gene amplicons corresponding to the Chloroflexota 631 ASVs were aligned using SINA online v.1.2.11 (Pruesse et al. 2007), and inserted into a Maximum Likelihood RAxML phylogenetic tree (Fig. 2a), using the maximum parsimony algorithm and selecting the best tree among 100 replicates on ARB (Ludwig et al. 2004).

For network analysis, the corresponding 16S rRNA gene dataset (i.e. 631 ASVs) was filtered to remove poorly represented ASVs and reduce network complexity by applying Spearman correlation (coefficient >0.6; *P* value <0.01), resulting in 128 ASVs for network construction after quality filtering. The non-random co-occurrence of the core community and community modularity were detected via a *walktrap* algorithm according to internal ties and their tie patterns between clusters, using the *igraph* package v. 1.2.5 (Csardi and Nepusz 2005) and *vegan* package v. 2.5–6 (Oksanen et al. 2019) in RStudio (http://www.rstudio.com/).

To confirm taxonomic assignment of our MAGs to Chloroflexota, we extracted proteins of the RNA polymerase sigma factor (*RpoD*) and performed a phylogenetic analysis (Vuillemin et al. 2020). All ORFs annotated to this gene were aligned against their top two BLASTp hits in the NCBI database, using MUSCLE (Edgar 2004). Conserved regions of the alignments were selected using Gblocks 0.91b (Castresana 2000) with the following settings: allowing for smaller final blocks, gap positions within the final blocks, and less strict flanking positions (http://phylogeny.lirmm.fr/). Phylogenetic analysis of the conserved amino acid alignments of the predicted proteins (Fig. 2b) was conducted in SeaView version 5.0.5 (Gouy et al. 2010), using PhyLM maximum likelihood (Guindon et al. 2010), with BLOSUM62 as the evolutionary model and 100 bootstrap replicates.

**Figure 3.**
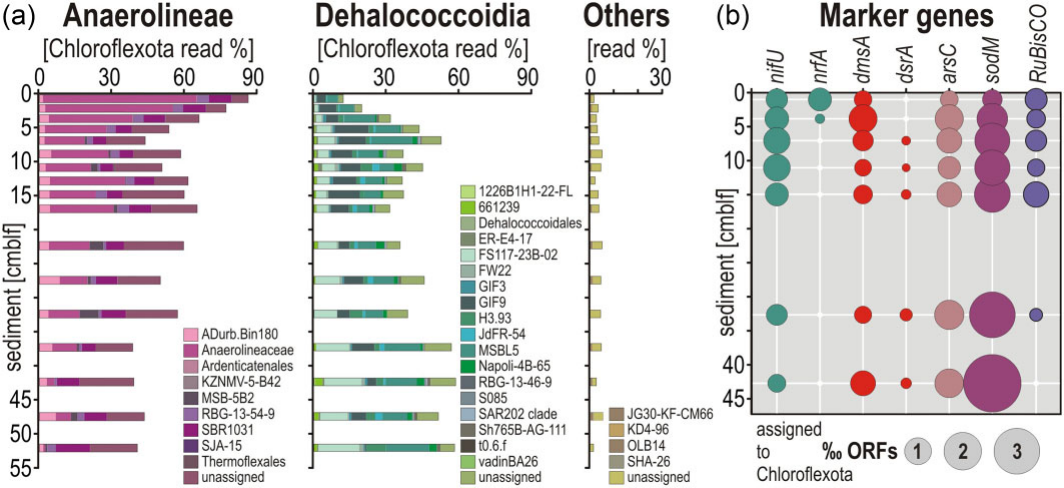
Relative abundances of Chloroflexota candidate clades and the main functional marker genes related to respiration types. (a) Relative abundances of 16S rRNA reads assigned to classes Anaeorolineae, Dehalococcoidia, and other candidates. The taxonomic assemblage shows a clear transition ∼7 cmblf, which is followed by a respective decrease and increase in abundances of Anaerolineae and Dehalococcoidia. (b) Specific functional marker genes show metabolic potential for ammonia-forming nitrite reduction (*nrfA*) in the upper 5 cmblf and dissimilatory sulfate reduction (*dsrA*) below. In addition, the concomitant dimethyl sulfoxide reductase (*dmsA*) and arsenate reductase (*arsC*) indicate metabolic potential for reduction of alternative electron acceptors. The presence of superoxide dismutase (*sodM*) in anaerobic Chloroflexota suggests defense mechanisms against oxidative stress, while nitrogen fixation proteins (*nifU*) imply an undetermined nitrogenase-oriented metabolism, likely functioning in ammonification. The large subunit of the form I ribulose-1,5-biphosphate carboxylase (*RuBisCO*) is not associated with any autotrophic pathway.

**Figure 4.**
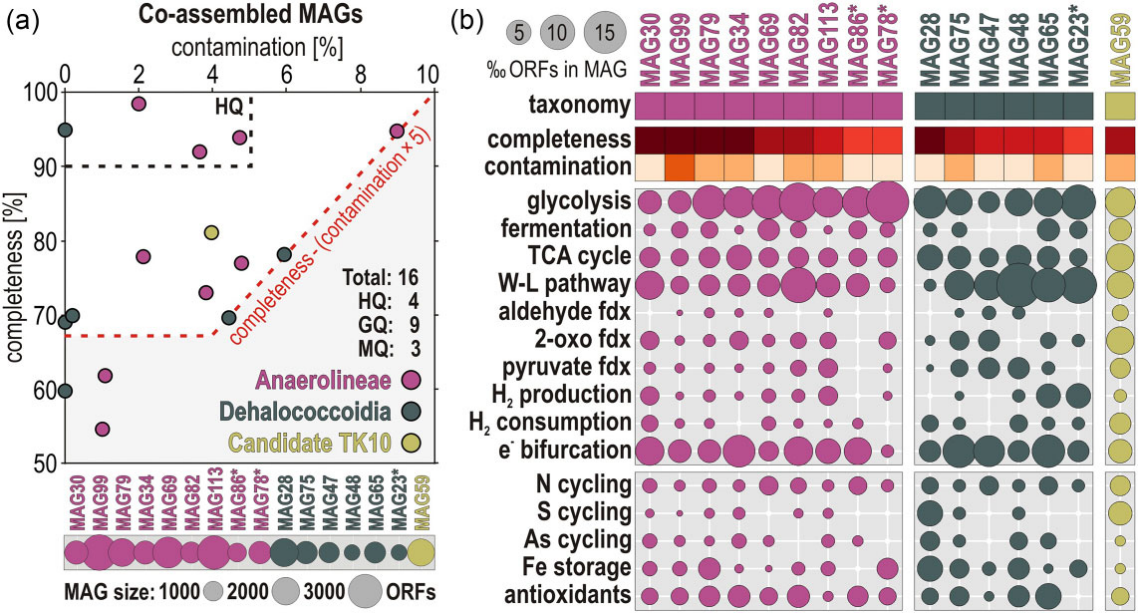
Quality of metagenome-assembled genomes (MAGs) and metabolic potential related to energy metabolisms and biogeochemical cycles. (a) Co-assembled MAGs plotted in terms of degree of contamination (*x*-axis) and completeness (*y*-axis). The 4 MAGs above the upper dashed line are of high quality (≥90% completeness; ≤5% contamination) and the 9 MAGs above the lower dashed line are of good quality (≥70% completeness; ≤10% contamination). MAGs marked with an asterisk (*) are of medium quality. (b) Bubble plot (‰ ORFs in the corresponding MAG) displaying the metabolic potential of each of the 16 MAGs assigned to Chloroflexota in terms of carbon assimilation [e.g. glycolysis, TCA cycle, Wood–Ljungdahl (W–L) pathway], hydrogen production and consumption, and biogeochemical cycling of nitrogen, sulfur, and metals. The full list of enzymes and their gene abbreviations included in these categories is available as Supporting Information (Supplementary Table S1).

**Figure 5.**
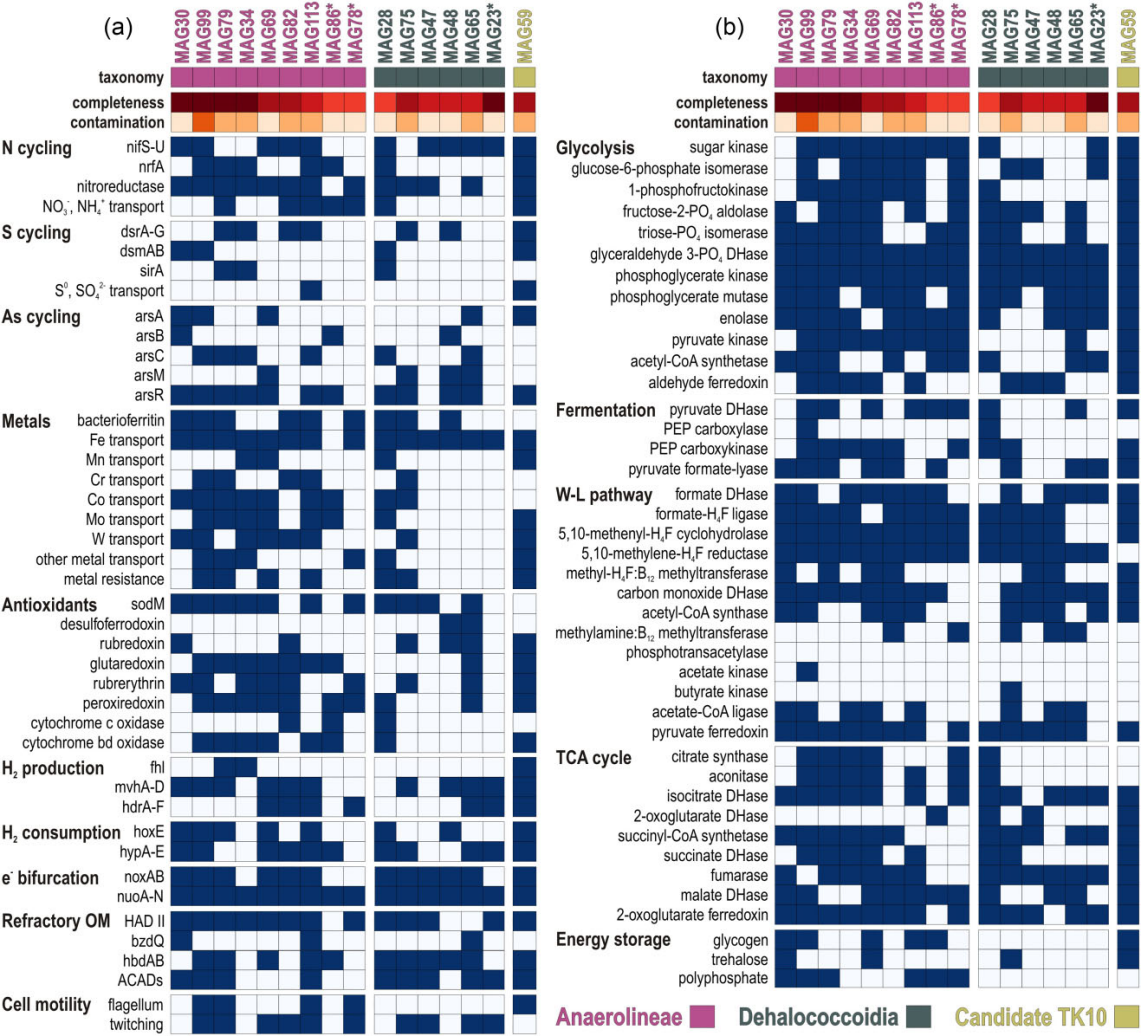
Specific metabolic traits identified in the 16 MAGs assigned to Chloroflexota. Presence/absence of functional marker genes in the 16 MAGs related to: (a) nitrogen (N), sulfur (S), arsenic (As), and metal cycling, antioxidant oxygen sensors, hydrogen (H_2_) production and consumption, electron (e^−^) bifurcation, degradation of refractory OM and fatty acids via β-oxidation, and cell motility; (b) carbon assimilation pathways, namely anaerobic glycolysis, fermentation, the carbonyl-branch W–L pathway, TCA cycle, cell energy storage, and cell motility. We found no genomic evidence for the glyoxylate pathway or reductive TCA cycle. The full list of enzymes and their gene abbreviations is available as Supporting Information (Supplementary Table S1).

To establish the relationship between pore water geochemistry, microbial taxonomic, and functional diversity (Figs 3–5), we looked for ORFs encoding genes involved in different types of anaerobic respiration related to nitrogen, sulfur, and metal cycling (Vuillemin et al. 2022, Vuillemin 2023). These included nitrogen fixation (*nifS-U*), ammonia-forming cytochrome c552 nitrite reductase (*nrfA*) with polysulfide reductase (*nrfD*) in formate-dependent nitrite reduction to ammonia, dissimilatory sulfite reductase (*dsrA-G*), dimethyl sulfoxide reductase (*dsmAB*), glutaredoxin-dependent arsenate reductase (*arsC*), and arsenical transport and resistance mechanisms (*arsA, arsB, arsM*, and *arsR*), several metal transporters, and antioxidant systems (e.g. desulfoferredoxin, rubredoxin, glutaredoxin, rubrerythrin, and periredoxin) with superoxide dismutase (*sodM*). Iron-related genes were identified using the FeGenie bioinformatic tool (Garber et al. 2020).

The metabolic capacity to access refractory OM sources (e.g. haloacids, aromatic compounds, fatty acids) was confirmed by the presence of ORFs encoding haloacid dehalogenase (HAD-II), benzoyl-CoA reductase (*bzdQ*), 3-hydroxyacyl- and 3-hydroxybutyryl-CoA dehydrogenase (*hbdAB*), and acyl-CoA and butyryl-CoA dehydrogenase (*ACADs*). Potential for cell energy storage (e.g. polyphosphate, glycogen, trehalose) was encoded by polyphosphate kinase (*ppk1*), glycogen synthase (*gys*), phosphorylase (*pyg*), trehalose synthase (*treS*), and phosphatase (*tpp1*), along with carbon storage regulator (*csrA*) and gas vesicle proteins (*gvp*).

We looked for all protein-encoding genes involved in the successive steps of carbon assimilation pathways (Fig. 5b), namely glycolysis, fermentation, the acetogenic (i.e. carbonyl-branch) W–L pathway, and the TCA cycle and their respective ferredoxin oxidoreductases (i.e. aldehyde, pyruvate, and 2-oxoacid). Evidence for the ability to use molecular hydrogen included formate hydrogenlyase (*fhl*), (non-)reducing F420 coenzyme methyl-viologen hydrogenase (*mvhA-D*), and heterodisulfide reductase (*hdrA-F*), which could be coupled with flavin-based electron bifurcation, i.e. NADH-flavin (*noxA-B*) and NADH-ubiquinone (*nuoA-N*) oxidoreductase, to serve as an electron donor, i.e. NAD-reducing (*hoxE*) and [NiFe] (*hypA-E*) hydrogenase. In comparison to the *nuo* complex, the detection of energy-conserving hydrogenase (*Ech*) and ion-translocating oxidoreductase (*Rnf*), respectively involved in the proton and sodium pump membrane complex, was minor. Finally, we also identified few ORFs encoding ribulose-1,5-biphosphate carboxylase (*RuBisCO*). Finally, we performed phylogenetic analysis of several of the corresponding amino acid sequences, using SeaView as described above, selecting ORFs with relevance to sulfur, arsenic, nitrogen, and carbon cycling, as well as antioxidant systems (Supplementary Figs S1–S4).

Phylogenetic analysis of the MAG taxonomy was performed by concatenating 16 ribosomal protein markers (Supplementary Fig. S5), using open scripts (Graham and Tully 2018), using the closest representative MAGs available from the GTDB database (Parks et al. 2022) as references. An anvi'o pangenome analysis (Eren et al. 2015) was carried out on the 16 MAGs of Chloroflexota with 18 representative MAGs from the GTDB database (Parks et al. 2022) to enable comprehensive alignment of gene contents across clades. Analysis of carbohydrate-active enzymes (CAZymes) was performed against the CAZy database (Cantarel et al. 2009; http://www.cazy.org/) integrated in anvi'o (Eren et al. 2015).

## Results

### Geochemical conditions at the sediment-water interface

The Conductivity–Temperature–Depth (CTD) profile shows dissolved O_2_ concentrations of 250 µM in surface waters starting to decrease below 50 m water depth (mwd) and reach detection limit ∼130 mwd. Values remain at that level down to the lake floor (Fig. 1a). Lake Towuti is thus stratified, with an oxycline occurring between 90 and 130 mwd and a pronounced monimolimnion. The pH profile displays a general decrease from 8.4 in surface waters to 7.0 at the sediment water interface (SWI). The main decrease in pH is concomitant with that of dissolved O_2_ concentrations through the oxycline (Fig. 1a). Analyses of discrete water samples show that dissolved Fe^2+^ concentrations increase below 130 mwd and, consistent with the dissolution of particulate Fe oxides in the oxycline (Bauer et al. 2020), gradually reaching 8 µM in the monimolimnion. The geochemical gradient further suggests that anoxic conditions at the SWI allow pore water Fe^2+^ to diffuse out of the sediment into the water column. SO_4_^2−^ concentrations in the water column are 20.2 (± 0.7), 11.9 (± 0.8), and 11.8 (± 0.2) µM at 60, 153, and 200 mwd, respectively (Vuillemin et al. 2016), consistent with anoxic ferruginous conditions in the bottom water.

### Subsurface biosphere: pore water metabolites, sulfate reduction rates, and cell count

In the sediment, the reduction of reactive ferric phases (e.g. ferrihydrite) leads to substantial release of Fe^2+^ to the pore water, as inferred from the increase in dissolved Fe^2+^ concentrations from 17 µM at the SWI to 49 µM at 32.5 cmblf (Fig. 1b). Pore water SO_4_^2−^ concentrations decrease from 9 µM at the SWI to ∼5 µM (detection limit: 2.0 µM) at 14 cmblf, with minor fluctuations in the remainder of the core. NH_4_^+^ concentrations increase from 20 µM at the SWI to 60 µM at 40 cmblf. Because NO_3_^−^ concentrations were below detection (<4.1 µM) throughout the sediment core, we consider microbial breakdown of OM as the main source of NH_4_^+^ released into the pore water. Methane concentrations increase gradually from 0 at the SWI to 550 µM at 50 cmblf. The fact that the methane profile is concave up indicates continuous *in-situ* production of methane with upward diffusion (Fig. 1b).

Despite a dearth of SO_4_^2−^ in both the monimolimnion (<12 µM) and pore water (<10 µM), radiotracer incubation experiments revealed measurable sulfate reduction rates (SRR) in the upper 50 cmblf. At the SWI, SRR are ∼10 nmol × cm^−3^ day^−1^ and decrease to ca. 2 nmol × cm^−3^ day^−1^ in the upper 5 cmblf. Below this depth, SRR fluctuates ∼1 nmol × cm^−3^ day^−1^ (Fig. 1b). Total cell counts (Fig. 1b) display a logarithmic decrease within the upper 55 cmblf (log_10_ = 8.4 to 7.8). In comparison, total cell counts normalized to the relative 16S rRNA gene abundance of Chloroflexota show a decrease in population density by one additional order of magnitude within the upper 5 cmblf (log_10_ = 7.8 to 6.7), and constant values below. Although SYBR Green I does not discriminate between living and dead cells, by binding to double-stranded DNA, SYBR Green I preferentially stains intact cells.

### Chloroflexota populations: 16S rRNA genes, alpha and beta diversities

The partial 16S rRNA gene phylogenetic tree (Fig. 2a) reveals 268 amplicon sequence variants (ASVs) in the class Dehaloccoidia, the clade GIF9 (60 ASVs) and MSBL5 (57 ASVs) being the most diverse. In the class Anaerolineae (328 ASVs), the family *Anaerolineaceae* (124 ASVs) and clade SBR1031 (71 ASVs) are the most diverse. The last 35 ASVs are distributed across minor classes of Chloroflexota (e.g. Chloroflexia, Ktedonobacteria). The same was observed in terms of their relative abundance and richness (Fig. 1c), the clades GIF9 and MSBL5 prevailing among the class Dehalocccoidia and the *Anaerolineaceae*, and the clade SBR1031 and unassigned groups prevailing among the class Anaerolineae (Fig. 3a). Other classes accounted for <10% of the Chloroflexota populations. The 50 most abundant ASVs represented ∼10% of all 16S rRNA genes sequenced at the SWI, decreasing to 5% in bottom sediments. These ASVs were mainly attributed to the order Anaerolineales and Aggregatilineales in the class Anaerolineae, and the clade MSBL5 and GIF9 in the class Dehalococcoidia (Supplementary Fig. S6).

The results of the PCoA indicate that the statistical distribution of both ASVs and ORFs assigned to Chloroflexota follows a depth trend in terms of taxonomic and functional diversity (coordinate 1), with a transition ∼15 cmblf inferred to reflect the transition from the sulfate reduction to the fermentative zone, in link with increasing pore water concentrations of metabolic end products (coordinate 2). Based on 631 ASVs, the principal coordinates 1 and 2 account for 37.8% and 16.7% of the taxonomic variation explained, respectively (Fig. 1d). The same stands true in terms of functional diversity predicted from 18 915 ORFs assigned to Chloroflexota. With principal coordinates 1 and 2, respectively accounting for 41.9% and 19.5% of the variation explained, this second PCoA overlaps well with the first (Fig. 1d).

The 128 ASVs used for network analysis contributed to ∼20% of the total population abundance in surface sediments (Supplementary Fig. S7). The species network consisted of 128 nodes (each node representing 1 ASV) and 2838 undirected edges. The density was 0.3492, the average path length was 1.3638 edges with a diameter of 3 edges, and the average clustering coefficient transitivity was 0.6885. The network analysis highlighted the importance of Dehaloccoidia clade GIF9, Anaerolineaceae (including clade SBR1031 and RBG-13–54–9), and Dehaloccoidia clade MSBL5 in clusters 1, 2, and 3, respectively.

### Population partitioning: predicted functions in metagenome-assembled genomes

In the metagenomes, we looked for functional genes (Supplementary Table S1) assigned to Chloroflexota, specifically for those related to respiratory types, and those either involved in nitrogen (*nifU*) or carbon fixation (*RuBisCO*), and determined their relative abundances, i.e. total number of ORFs assigned to Chloroflexota per separate metagenome (‰ ORFs). We found that specific respiratory functions show a depth trend parallel to the taxonomic variation observed among Chloroflexota populations (Fig. 3a–b). For instance, metabolic capacity to reduce nitrite to ammonium (*nrfA*) is only present in the uppermost sediments, whereas dissimilatory sulfite reduction to sulfide (*dsrA*) is only detectable below 7 cmblf. Metabolic potential for the reduction of dimethyl sulfoxide to dimethyl sulfide (*dmsA*) is present throughout the sediment sequence, but greatest at 3 cmblf, which is also the case for the reduction of arsenate to arsenite (*arsC*). The relative abundance of ORFs assigned to superoxide dismutase (*sodM*), which increases with sediment depth (Fig. 3b), is rather related to antioxidation and redox sensors, not to aerobic respiration (Lancaster et al. 2004, Johnson and Hug 2019). Metabolic potential for nitrogen fixation (*nifU*) was equally present along the entire core, but is indicative of a redox function (Oshiki et al. 2022, Bovio-Winkler et al. 2023) in link to nitrite reduction to ammonium (i.e. *nrf* pathway). The number of ORFs annotated as *RuBisCO* large subunits (Supplementary Fig. S1) decreased with sediment depth (Fig. 3b), which may indicate different adaptation to CO_2_ concentrations (Banda et al. 2020) rather than any metabolic potential for autotrophic dark carbon fixation via the Calvin-Benson-Bassham (CBB) pathway.

From *de novo* processing of combined metagenomic reads, we obtained 16 MAGs assigned to Chloroflexota (Fig. 4a), of which 4 were of high quality (≥90% completeness; ≤5% contamination), 9 could be considered of good quality (≥70% completeness; ≤10% contamination), and 3 were of medium quality (≥50% completeness; ≤10% contamination). We identified nine MAGs belonging to the class Anaerolineae, six MAGs to the class Dehaloccoidia, and one MAG to the candidate clade TK10 (Supplementary Table S2). These taxonomic assignments were further confirmed by a phylogenetic analysis of RNA polymerase sigma factor (*RpoD*) proteins extracted from 13 MAGs (Fig. 2b), and 16 concatenated ribosomal proteins extracted from all 16 MAGs (Supplementary Fig. S5). The overall genetic content of the MAGs shows that metabolic processes involved in OM remineralization, nitrogen, sulfur, and metal biogeochemical cycles are differently distributed among the classes Anaerolineae and Dehalococcoidia (Fig. 4b). The relative abundance of ORFs per MAG (‰ ORFs) suggests that both classes have high metabolic capacity for anaerobic glycolysis and a lesser one for fermentation. Further, MAGs in the class Anaerolineae display higher potential to couple glycolysis to the tri-carboxylic acid (TCA) cycle, whereas the Dehalocccoidia lean towards the W–L pathway with the use of molecular hydrogen. Both classes can perform flavin-based electron bifurcation with NADH. In both cases, antioxidant systems and iron storage are important features of the MAGs. Finally, the MAG assigned to the candidate clade TK10 apparently displays high versatility with respect to the aforementioned metabolic features (Fig. 4b).

### Selective metabolic traits: genetic content of metagenome-assembled genomes

To assess prominent metabolic features acting on OM remineralization, energy production, and carbon assimilation pathways in the MAGs of Chloroflexota, we listed the presence/absence of all the corresponding ORFs (Supplementary Table S1) with specific focus on each successive step of glycolysis, fermentation, the acetogenic (i.e. carbonyl-branch) W–L pathway, and TCA cycle (Fig. 5a–b).

**Figure 6.**
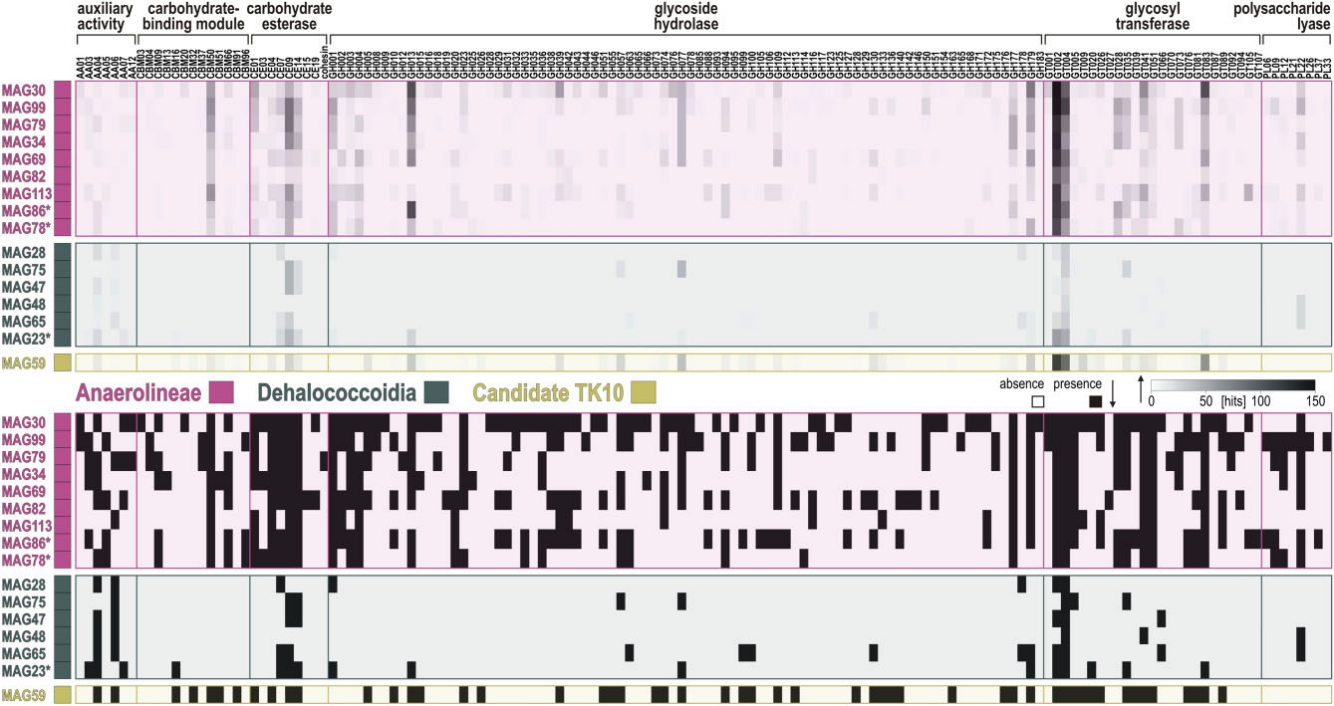
CAZymes identified in the 16 MAGs assigned to Chloroflexota. Heatmaps of the CAZymes analysis performed on the 16 partial MAGs assigned to Chloroflexota against the CAZy database, according to the number of hits (**top**) and presence/absence (**bottom**) of CAZy families. Enzymatic names and targeted substrates corresponding to each CAZy family are available as Supporting Information (Supplementary Table S3).

In brief, MAGs assigned to Anaerolineae show many genes involved in nitrogen cycling, metal transporters (e.g. Fe, Mn, Cr, Mo, W, As, Co, Cd, and Zn), and antioxidant redox systems (e.g. glutaredoxin, rubrerythrin, and periredoxin) functioning with superoxide dismutase (*sodM*) (Johnson and Hug 2019). The presence of the cytochrome cbb3 and cytochrome bd oxidase in several MAGs argues for a certain degree of facultative microaerobic processes (Fig. 5a), with metabolic respiratory potential to reduce nitrite to ammonium (*nrfA, nrfD*, and *nifS-U*) and dimethyl sulfoxide (*dsmAB* and *dsrC-E*), whereas arsenate is not respired (Saltikov et al. 2003) but reduced with glutaredoxin as electron carrier (*arsC*) and the produced arsenite detoxified (*arsA, arsB, arsM*, and *arsR*). Specific organic substrates to which the genetic content of MAGs would enable access include nitro functional groups (nitroreductases), haloacids (HAD-II), benzoate (*bzdQ*), and fatty acids (*hbdAB* and *ACADs*). Anaerolineae also have the cellular capacity to store energy in the form of glycogen (*gys* and *pyg*), trehalose (*treS* and *tpp1*), and polyphosphate (*ppk1*). Proteins related to gas vesicles (*gvp*) and carbon storage (*crsA*) were identified in several MAGs (i.e. MAG82, MAG99, and MAG113). Finally, the large subunit of *RuBisCO* was detected in MAG30 only (Supplementary Fig. S2), which is closely affiliated with the candidate order *Aggregatilineales* described as facultative anaerobes (Nakahara et al. 2019). Metabolic potential related to iron processes includes iron acquisition and regulation, without evidence of dissimilatory reduction and/or oxidation (Supplementary Fig. S8). In addition, Anaerolineae exhibit a high diversity of CAZymes (Fig. 6), with numerous hits to specific families among, e.g. carbohydrate-binding modules (i.e. CBM50), carbohydrate esterases (i.e. CE1, CE7, CE9, and CE14), glycoside hydrolases (i.e. GH1-4, GH13, GH39, GH76-77, and GH177-179), glycosyl transferases (i.e. GT2, GT4, GT28, GT35, GT41, and GT83), and polysaccharide lyases (PL22). The substrates targeted by the corresponding enzymes (Zheng et al. 2023; Supplementary Table S3) suggest that Anaerolineae have metabolic potential to oxidize diverse carbohydrates derived from complex polymers (e.g. chitin, cellulose, and glycogen) and to assimilate their derivatives (e.g. carboxyl ester, carboxamide, glycosyl, and ketose), producing acetate as a by-product of glycolysis (Bovio-Winkler et al. 2024). Genetic evidence for cell motility included flagellum biosynthesis, gliding, and twitching motility (Fig. 5), suggesting both planktonic and benthic lifestyles.

The genetic content of MAGs assigned to Dehalococcoidia points to a benthic fermentative lifestyle with several hydrogenases involved in molecular hydrogen production (*fhl, mvhA-D*, and *hdrA-F*) and consumption (*hoxE* and *hypA-E*) coupled to energy conservation via flavin-based electron bifurcation (*noxA-B* and *nuoA-N*), and an acetogenic (carbonyl-branch) W–L pathway and a partial TCA cycle (Fig. 5). We also identified ORFs encoding methanol and 1, 2, 3-methylamine: corrinoid methyltransferase (*mta, mtm, mtb*, and *mtt*) in three MAGs (Fig. 5b), which are enzymes normally restricted to the methyl-branch of the archaeal W–L pathway (Adam et al. 2018). Metabolic potential for β-oxidation of fatty acids is present (*hbdAB, ACADs*). In comparison, metabolic potential for VFA kinases is absent (*akn*) or negligible (*bkn*), implying that there can be no acetate conversion for energy production via SLP. Metabolic potential involving CAZymes appears to be quite limited (Fig. 6), and targets in particular partially oxidized compounds (LaRowe and Van Cappellen 2011), such as alcohols, phenols, ketones, and carboxylic acids (e.g. AA4, AA6, CE7, CE9, CE14, GT2, and GT4). In terms of oxidants, there is no evidence for dissimilatory iron reduction and/or oxidation, although metabolic potential for iron acquisition, storage, and regulation is present (Supplementary Fig. S8). Detection of *nrfA, dsmAB*, and *arsC* in MAGs of Dehalococcoidia appears limited (Fig. 5a) and not particularly consistent (Supplementary Figs S2–S3). Although we identified desulfoviridin in MAG75 (i.e. *Dehalococcoidales*), we mainly identified the subunit E of the *dsr* operon (Supplementary Fig. S2), which is reported to act in intracellular sulfur reduction and assimilation (Venceslau et al. 2014), currently limiting the evidence for dissimilatory sulfite reduction in Dehalococcoidia (Wasmund et al. 2017). The only MAG including metabolic potential for dissimilatory sulfate reduction (*dsrAB*) is attributed to the candidate clade TK10 (Supplementary Fig. S2), which exhibits high metabolic versatility and has been suggested to be a hypolimnion-specific planktonic clade (Mehrshad et al. 2018b), which the presence of genes encoding a flagellum currently supported (Fig. 5a).

In terms of carbon assimilation pathways, we noticed the complete absence of ORFs encoding phosphotransacetylase and acetate kinase (Fig. 5b) and detected only a few encoding butyrate kinase (Ferry 2011) in all MAGs of Anaerolineae, Dehalococcoidia, and candidate TK10. This suggests that none of these Chloroflexota generate one additional ATP from the conversion of acetyl-CoA to acetate (i.e. homoacetogenesis) or run the reverse acetoclastic reaction via SLP to convert acetate into acetyl-CoA (Müller 2003, Ragsdale and Pierce 2008, Schuchmann and Müller 2016). Instead, the detection of ORFs encoding acetate-CoA ligase (*acss2*) suggests that acetate is commonly incorporated into cell biomass. Similarly, only a few ORFs encoding phosphoenolpyruvate (PEP) carboxylase, a main enzymatic step in lactate/pyruvate fermentation, were detected while ORFs encoding pyruvate formate-lyase (*pfl*), also known as formate C-acetyltransferase (Crain and Broderick 2014), were identified in 10 MAGs from all 3 classes.

The pangenome analysis provided definitive evidence of the metabolic divergence between MAGs belonging to the classes Anaerolineae and Dehalococcoidia (Supplementary Fig. S9). We acknowledge that the size and completeness of the MAGs assigned to Dehalococcoidia are generally lesser than those assigned to Anaerolineae (Fig. 4a) and that the absence of specific marker genes may result from the degree of MAG completeness and limited sequencing depths. The full list of enzymes and gene abbreviations is available as Supporting Information (Supplementary Table S1). Functional annotations of specific proteins were confirmed via phylogenetic analysis of the corresponding amino acid sequences (Supplementary Figs S1–S4).

## Discussion

### Anaerolineae exhibit versatile use of alternative electron acceptors

Ferruginous Lake Towuti experiences depletion of the most favorable electron acceptors (i.e. O_2_, NO_3_^−^, Mn^4+^, Fe^3+^, and SO_4_^2−^) already in its bottom waters (Fig. 1a) and even further in the pore water at shallow sediment depths (Vuillemin et al. 2016). Ferric minerals in the sediment, mostly detrital goethite, are poorly reactive towards microbial reduction (Friese et al. 2021). Nevertheless, some Chloroflexota exhibited metabolic potential for ferrous iron acquisition, regulation, and storage (Supplementary Fig. S8) with several metalloenzymes (Fig. 5a) that could catalyze redox reactions, either via electron transfer from less reactive ferric phases, or via electron bifurcation during fermentation, or both (Hay Mele et al. 2023).

Several MAGs among Anaerolineae displayed metabolic versatility in both microaerophilic and anaerobic respiration pathways (Fig. 5a), taking advantage of alternative electron acceptors. These included nitrite (*nrfA*) with cytochrome c552, arsenate (*arsC*) with glutaredoxin, and dimethyl sulfoxide (*dmsAB*) combined with the *dsrC-E* (Supplementary Fig. S3) as carriers for intracellular sulfur reduction (Venceslau et al. 2014). Despite the absence of *dsrAB* genes in Anaerolineae, this suggested an intricate respiration of metabolic by-products derived from the canonical chain of electron acceptors (i.e. NO_3_^−^, Fe^3+^, and SO_4_^2−^). Furthermore, the many metalloproteins identified (i.e. desulfoferredoxin, rubredoxin, glutaredoxin, rubrerythrin, and periredoxin) could function as antioxidant systems with the superoxide dismutase (Sutherland et al. 2021), and redox cofactors in the electron transport chain with cytochrome cbb3 (Esposti 2020), cytochrome bd (Giuffrè et al. 2014), as well as in fermentation with flavin-based electron bifurcation (*noxAB* and *nuoA-N*). The MAGs of Anaerolineae also contained a plethora of metal transporters (e.g. Fe, Mn, Cr, Mo, W, As, Co, Cd, and Zn) and exporters providing resistance to heavy metals, as well as iron storage mechanisms (i.e. ferritin, bacterioferritin), which are essential for the synthesis of the identified cytochromes (i.e. cbb3, bd, and c552), metal clusters, and organometallic complexes involved in the acetogenic W–L pathway (Ragsdale 2008). The metabolic capacity to synthesize storage compounds (i.e. glycogen, trehalose, and polyphosphate) from excess energy was also present across several MAGs of Anaerolineae (Fig. 5a), and has been reported as a “feast-or-famine” adaptive feature at the transition between aerobic (i.e. storage) and anaerobic (i.e. consumption) conditions (Speirs et al. 2019, Liu et al. 2022, Bovio-Winkler et al. 2023, Petriglieri et al. 2023). Consistently, the metabolic potential related to CAZymes (Fig. 6) would enable them to oxidize various sugars and polymers (i.e. feast) and subsequently utilize their derivatives and stored glycogen (i.e. famine), releasing organic acids, and acetate as by-products (LaRowe and Van Cappellen 2011).

The rapid decrease in the relative abundance of Anaerolineae underneath the SWI (Fig. 1c) constituted further evidence of their preference for microaerobic habitats, such as Lake Towuti’s oxycline and ferruginous bottom waters, and revealed their limited ability to survive deeper into the fermentative zone. In contrast, Dehalococcoidia appeared to specialize in (homo)acetogenic fermentations in anoxic sediments (e.g. clade GIF9 and MSBL5), evolving as slow-growing but efficient scavengers of organic acids and hydrogen (Ziv-El et al. 2012).

### Dehalococcoidia present strategic shortcuts in carbon assimilation pathways

The acetogenic W–L pathway is linked to energy conservation alongside fermentation (Lever 2012), converting glucose into acetate for biomass synthesis, and for energy by converting acetyl-CoA into acetate via phosphotransacetylase (*pta*) and acetate kinase (*akn*) (Müller 2003, Schuchmann and Müller 2016). Autotrophic CO_2_ reduction with molecular hydrogen via homoacetogenesis produces an additional acetate (Ragsdale 2008, Ferry 2011). Although the reversible *pta*-*akn* reaction that can produce or dissimilate acetate (Ragsdale and Pierce 2008) is energetically more favorable, the W–L pathway observed in Dehalococcoidia and Anaerolineae (Fig. 5b) only includes the non-reversible assimilatory acetate-CoA ligase (AMP-forming) pathway (Wolfe 2005). Acetogenic fermentation in these Chloroflexota can apparently be combined with a variety of electron donors in Dehalococcoidia, and alternative electron acceptors (e.g. nitrite, dimethyl sulfoxide, and arsenate) as electron sinks in Anaerolineae for redox balancing (Müller 2003).

For energy conservation in acetogenic CO_2_ fixation (Mostafa et al. 2022), Dehalococcoidia could use molecular hydrogen as the reductant during fermentation via their hydrogenases (i.e. *mvhA-G* and *hdrA-F*) and harness redox power via electron-bifurcating flavoproteins (Buckel and Thauer 2018) coupled to NADH (*nuoA-N*), NADPH (*noxA-B*), and ferredoxins. In addition, they exhibited ORFs encoding different methylamine corrinoid methyltransferases (Fig. 5b) whose proposed role is to catalyze the one-step reduction of CO_2_ to CO (Sewell et al. 2017) and make the carbon monoxide dehydrogenase (*codh*) of the W–L pathway carbonyl-branch more efficient under high CO_2_ conditions (Adam et al. 2018). Strikingly, phosphotransacetylase was not detected in any of our 16 MAGs, and we identified very few ORFs encoding acetate kinase or butyrate kinase (Fig. 5b). This suggested that the carbonyl-branch of the W–L pathway in these Chloroflexota mainly assimilates acetate via the acetate-CoA ligase, which yields less energy than SLP.

The detection of 3-hydroxyacyl- and 3-hydroxybutyryl-CoA dehydrogenase (*hbdAB*) and acyl-CoA and butyryl-CoA dehydrogenase (*ACADs*) indicated that Dehalococcoidia could preferentially perform β-oxidation of fatty acids into CO_2_ and acetyl-CoA rather than SLP of VFAs (Folch et al. 2021). Although limited, their metabolic potential for CAZymes (Fig. 6) could allow secondary fermentation of partially oxidized compounds (Bovio-Winkler et al. 2024), e.g. alcohols, phenols, ketones, and carboxylic acids (LaRowe and Van Cappellen 2011). The downstream PEP carboxylase, an essential enzymatic step in fermentative generation of acetyl-CoA for energy production in the TCA cycle, was barely detected. Instead, generation of acetyl-CoA could proceed via pyruvate dehydrogenase and pyruvate formate-lyase as well. Pyruvate formate-lyase is a similar way to supply the TCA cycle with acetyl-CoA during anaerobic glycolysis (Crain and Broderick 2014), yielding an additional formate that can be disproportionated in the acetogenic W–L pathway for synthesis of acetyl-CoA via the formate hydrogenlyase (*fhl*) and formate dehydrogenase (*fdh*) complex. Further, we identified ORFs encoding the benzoyl-CoA reductase (*bzdQ*), which anaerobically converts benzoate (and other aromatic carboxylic acids) into acetyl-CoA (Sewell et al. 2017).

Despite the multiple pathways in which acetyl-CoA could be generated, the mere absence of 2-oxoglutarate dehydrogenase could be expected to interrupt the oxidative reaction chain of the TCA cycle consuming acetyl-CoA. The replacing enzyme isocitrate lyase that forms succinate in the glyoxylate cycle to avoid CO_2_ production (i.e. decarboxylation) was neither detected. Instead, we identified haloacid dehalogenase (HAD-II), which acts on C_2_–C_4_ chain acids (Gaboyer et al. 2015, Wang et al. 2021) producing 2 hydroxy carboxylic acids that could be metabolized in the next step of the TCA cycle (Fig. 5b), namely succinate-CoA synthetase. The activity of this enzyme can be reversed for anabolic purposes when the 2-oxoglutarate route is heavily repressed during anaerobic growth, thereby replenishing the TCA cycle intermediates (i.e. anaplerosis) for energy production and “housekeeping” amino acid biosynthesis (Cronan and Laporte 2005, Inigo et al. 2021).

Altogether, the use of shortcuts in carbon assimilation pathways appeared consistent with the faculty of Dehaloccoidia to scavenge most of their required substrates from metabolites released by active or dying microorganisms, e.g. organic acids, haloacids, and fatty acids (Hug et al. 2012, Vuillemin et al. 2020), and combine their fermentation with CO_2_ fixation for the production of acetyl-CoA and biomass via an acetogenic W–L pathway. Finally, we identified specific ORFs encoding the large subunit of *RuBisCO* (Supplementary Fig. S2), which is not involved in the autotrophic CBB pathway (Banda et al. 2020, West-Roberts et al. 2021) but in fatty acid synthesis via the biotin subunit of the acetyl-CoA carboxylase. Although the *RuBisCO* large subunit without its small subunits is known to only operate in CO_2_-rich environments (Tabita et al. 2008, Badger and Sharwood 2023), the enzymatic functions of *RuBisCO*—like proteins (form IV) are not yet fully understood, whether they relate to CO_2_ attack or sulfur oxidative stress (Tabita et al. 2008, Banda et al. 2020, Prywes et al. 2023). Despite their detection in metagenomes (Fig. 3b; Supplementary Fig. S1), *RuBisCO* proteins were only assembled in one single MAG, which is phylogenetically close to a facultative anaerobe among the order *Aggregatilineales* (Nakahara et al. 2019).

### Metabolic traits inherited from ancient ferruginous ecosystems

Evolutionary studies on clades of Chloroflexota revealed a complex history of gene acquisition (Rao et al. 2022) that resulted in parallel pathways to exploit alternative electron acceptors, refractory dissolved OM, and relatively rare organic compounds (West-Roberts et al. 2021, Chiriac et al. 2023, Palmer et al. 2023). Among chemoorganotrophic Chloroflexota, the classes Anaerolineae and Dehalococcoidia (Fig. 2a) exhibit the strongest phylogenetic radiation within the phylum (Wiegand et al. 2023) that traces back to Earth’s ocean oxygenation (Shang et al. 2022). For instance, the early branching clade Dehalococcoidia SAR202 developed a slow metabolism (Saw et al. 2020) based on aerobic glycolysis inhibited by exposure to light (Lim et al. 2023), whereas anaerobes developed ways to store sugars (e.g. glycogen and trehalose), polyphosphates, and metals (e.g. ferritin and bacterioferritin) to survive periods of limited resources (Ayala-Muñoz et al. 2022, Petriglieri et al. 2023).

In Lake Towuti, Anaerolineae could thrive in anoxic ferruginous bottom waters and highly colonize the SWI, developing a specific niche in both planktonic (i.e. flagellum) and benthic (i.e. twitching) lifestyles. Their use of unconventional electron acceptors (i.e. *nrfA, dmsA*, and *arsC*) with different cytochromes (i.e. cbb3, bd, and c552) and multiple redox metalloproteins (e.g. rubredoxin, rubrerythrin, glutaredoxin, periredoxin, desulfoferredoxin, and *sodM*) enable them to cope with fluctuating oxygen levels in Lake Towuti’s bottom waters. These metabolic traits may represent ancestral features underlying energy-conserving catalytic reactions in primitive hydrothermal ecosystems (Reysenbach and Cady 2001, Wells et al. 2020, West-Roberts et al. 2021) and explain their wide geographic distribution from ferruginous deep waters (Chiriac et al. 2023, Schauberger et al. 2024) to sulfidic sediments (Vuillemin et al. 2022). In deeper sediments, the Dehalococcoidia evolve as benthic acetogens, fermenting various organic substrates to generate energy from acetyl-CoA and biomass from acetate, and bypassing specific steps in carbon assimilation pathways to efficiently scavenge metabolites (e.g. fatty acids, haloacids, and aromatic acids), which they use in secondary fermentation under limited OM availability, thus being able to colonize even the abyssal subseafloor (Vuillemin et al. 2020). Energy conservation during fermentation is achieved via acetogenic CO_2_ fixation, which is apparently restricted to the carbonyl-branch of the W–L pathway, with nevertheless the presence of methylamine: corrinoid methyltransferases inherited from a theoretical methanogenic ancestor (Adam et al. 2018) to enhance carbon monoxide dehydrogenase activity under high CO_2_ conditions (Sewell et al. 2017).

In conclusion, the respective metabolic versatility of these two classes enables them to use alternative electron acceptors and donors derived from canonical respiratory pathways and OM remineralization to cross-feed on specific metabolic by-products under ferruginous conditions, thereby partitioning Anaerolineae and Dehalococcoidia populations at the interface between the sulfate-reducing and fermentative zones.

## Supplementary Material

fiae140_Supplemental_File

## Data Availability

The raw sequencing data (16S rRNA libraries and metagenomes) are publicly available on the European Nucleotide Archive (ENA) via the project accession number PRJEB66721 at (https://www.ebi.ac.uk/). The geochemical data is publicly available as dataset #861 437 on the PANGAEA database (https://doi.pangaea.de/). All scripts, code, and instructions regarding how to conduct the BLASTp workflow for taxonomic and functional annotations are posted on GitHub with a link to the MetaProt page (github.com/williamorsi/MetaProt-database).
