# Supplementary material for: Taxonomic and functional partitioning of Chloroflexota populations under ferruginous conditions at and below the sediment-water interface
Source: FEMS Microbiol Ecol. 2024 Oct 9;100(12):fiae140. doi: 10.1093/femsec/fiae140 (PMC11650866; doi:10.1093/femsec/fiae140)
Supplement: fiae140_Supplemental_File [file fiae140_supplemental_file.pdf]

Supporting Information

Taxonomic and functional partitioning of Chloroflexota populations under ferruginous conditions at and below the sediment-water interface

Aurèle Vuillemin, Fatima Ruiz-Blas, Sizhong Yang, Alexander Bartholomäus,
Cynthia Henny, and Jens Kallmeyer

Content: Supplementary Figures (9); Supplementary Tables (3)

Supplementary Figures

- **Supplementary Figure S1.** Phylogenetic tree of marker genes related to specific carbon substrates
- **Supplementary Figure S2.** Phylogenetic tree of marker genes related to sulfur and arsenic cycling
- **Supplementary Figure S3.** Phylogenetic tree of marker genes related to nitrogen cycling
- **Supplementary Figure S4.** Phylogenetic tree of marker genes related to antioxidant systems
- **Supplementary Figure S5.** Phylogenetic tree of 16 concatenated ribosomal proteins
- **Supplementary Figure S6.** Relative abundances and phylogenetic tree of Chloroflexota top 50 ASVs
- **Supplementary Figure S7.** Network analysis of amplicon variant sequences assigned to Chloroflexota and phylogenetic trees of the corresponding 16S rRNA gene amplicons
- **Supplementary Figure S8.** Heatmaps of metabolic potential based on FeGenie iron processes
- **Supplementary Figure S9.** Pangenome analysis of the 16 metagenome-assembled genomes (MAGs) attributed to Chloroflexota with 18 representative MAGs from the GTDB database as references

Supplementary Tables

- **Supplementary Table S1.** List of enzymes and gene abbreviations for Open Reading Frames (ORFs)
- **Supplementary Table S2.** Statistics of *de novo* co-assembly for the 16 MAGs
- **Supplementary Table S3.** List of CAZyme families, names of enzymes and targeted substrates

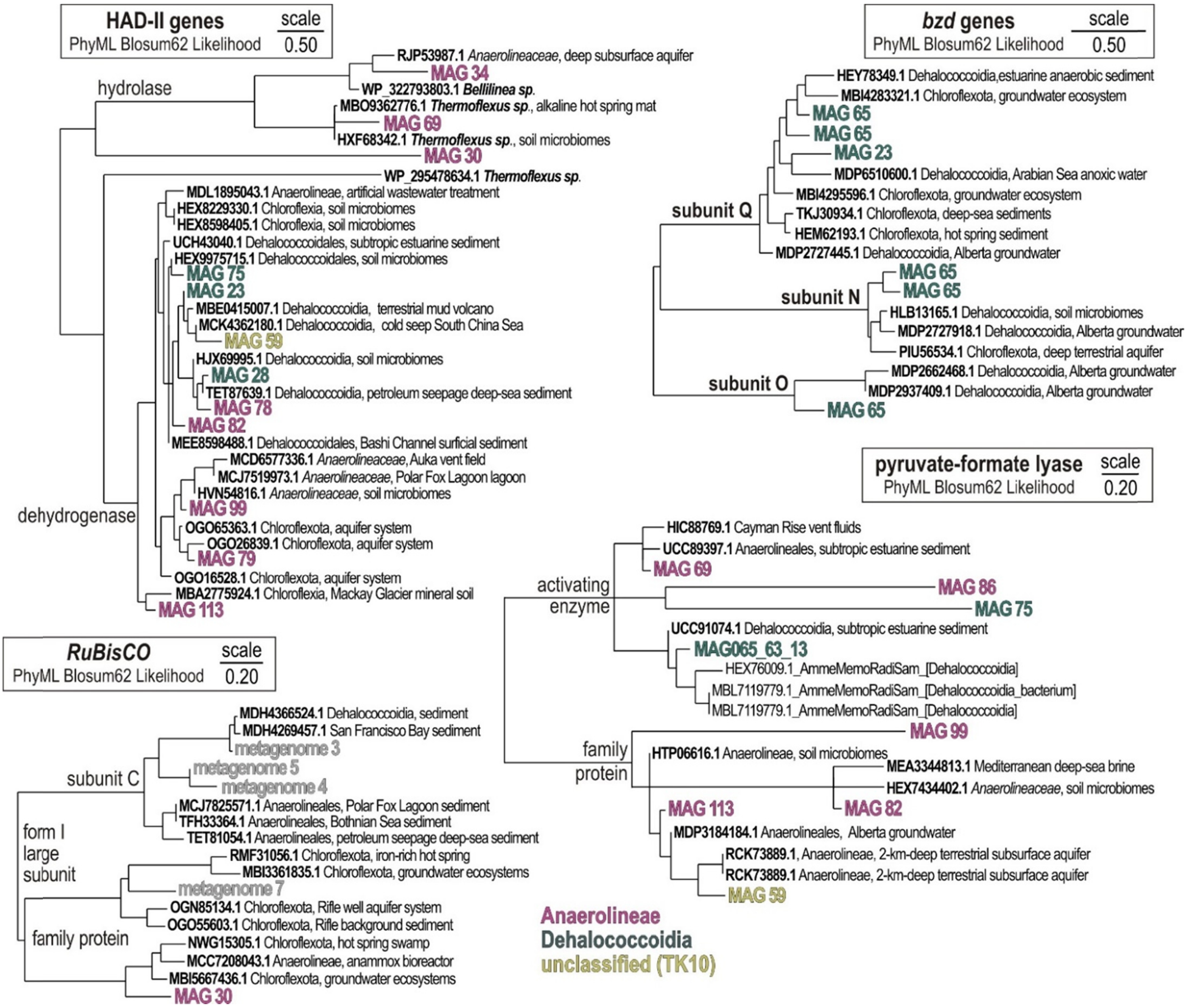

Supplementary Figure S1. Phylogenetic tree of marker genes related to carbon cycling. PhyML Blosum62 phylogenetic tree of conserved regions for extracted ORFs encoding the haloacid dehydrogenase (46 amino acids), benzoyl-CoA reductase (140 amino acids), pyruvate-formate lyase (37 amino acids) and ribulose-1,5-biphosphate carboxylase (*RuBisCO*, 286 amino acids) assigned to Chloroflexota based on 100 replicates. Boldface types signify cultivated species and NCBI sequence accession numbers.

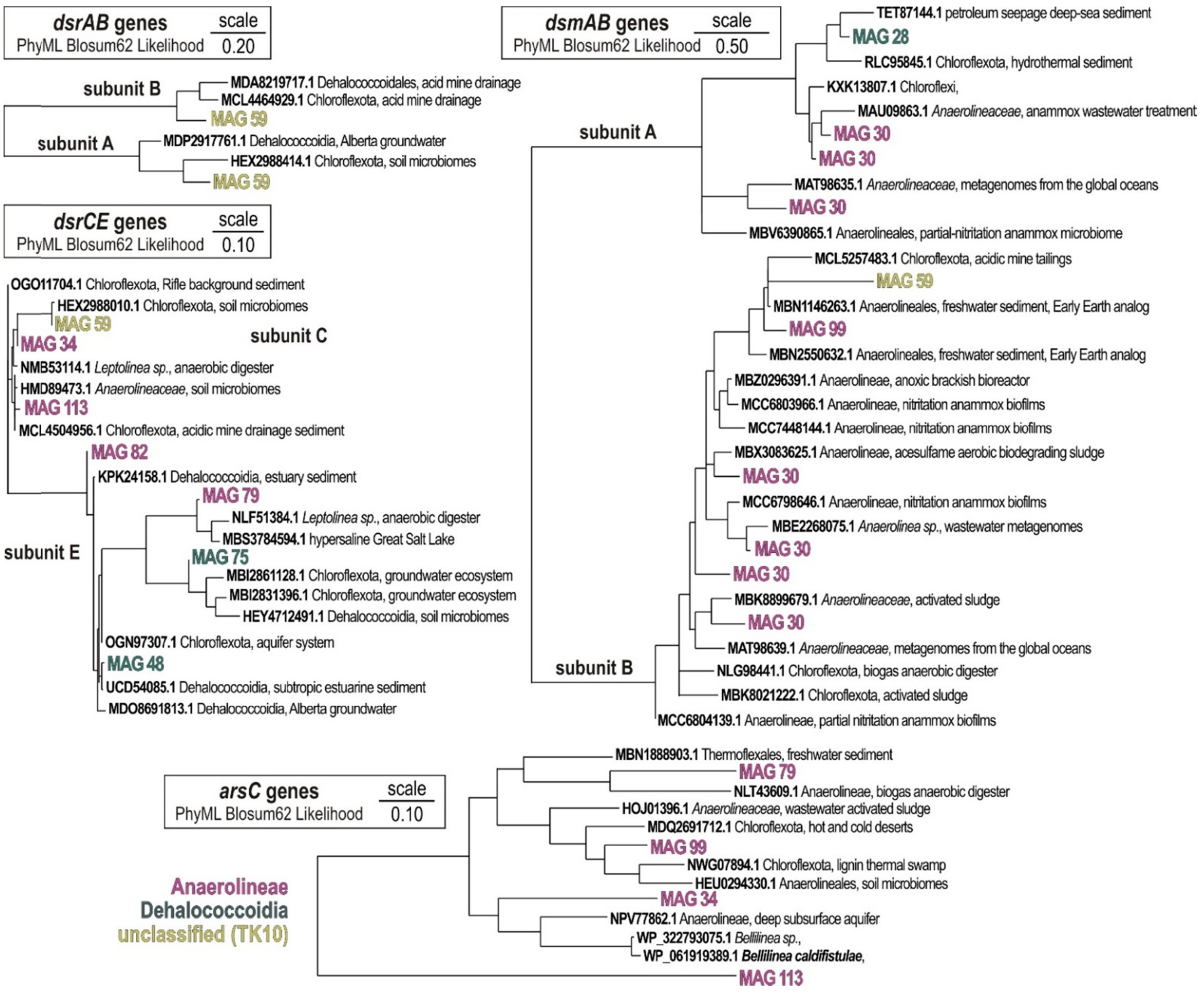

Supplementary Figure S2. Phylogenetic tree of marker genes related to sulfur and arsenic cycling. PhyML Blosum62 phylogenetic tree of conserved regions for extracted ORFs encoding the dissimilatory sulfate reductase subunit A and B (280 amino acids), C and E (38 amino acids), the anaerobic dimethyl-sulfoxide reductase subunit A and B (68 amino acids), and the arsenate reductase subunit C (130 amino acids) assigned to Chloroflexota based on 100 replicates. Boldface types signify cultivated species and NCBI sequence accession numbers.

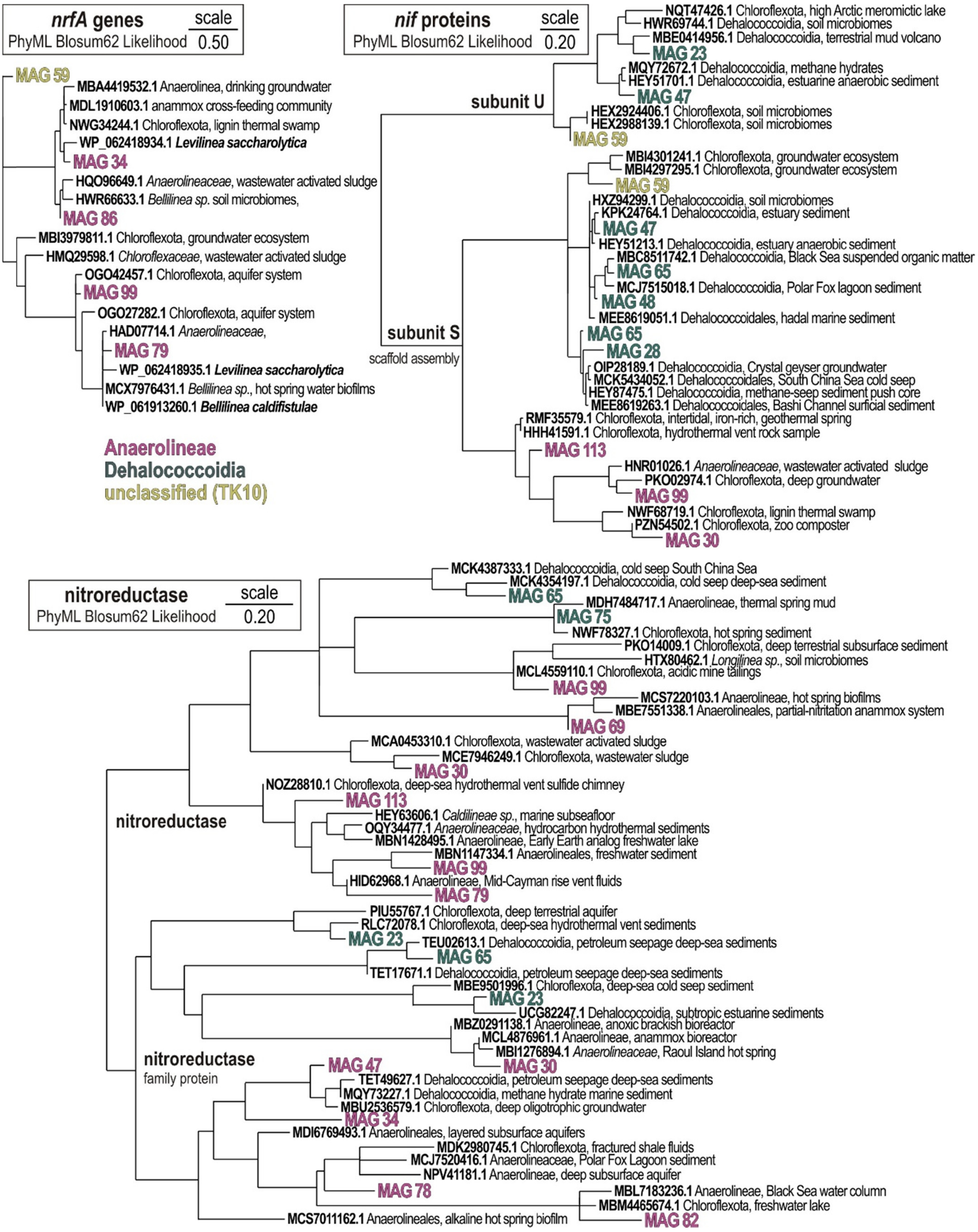

Supplementary Figure S3. Phylogenetic tree of marker genes related to nitrogen cycling. PhyML Blosum62 phylogenetic tree of conserved regions for extracted ORFs encoding the ammonia-forming cytochrome C nitrite reductase (*nrfA*, 36 amino acids), nitrogen fixation family proteins (*nifS-U*, 70 amino acids) and nitroreductase proteins (29 amino acids) assigned to Chloroflexota based on 100 replicates. Boldface types signify cultivated species and NCBI sequence accession numbers.

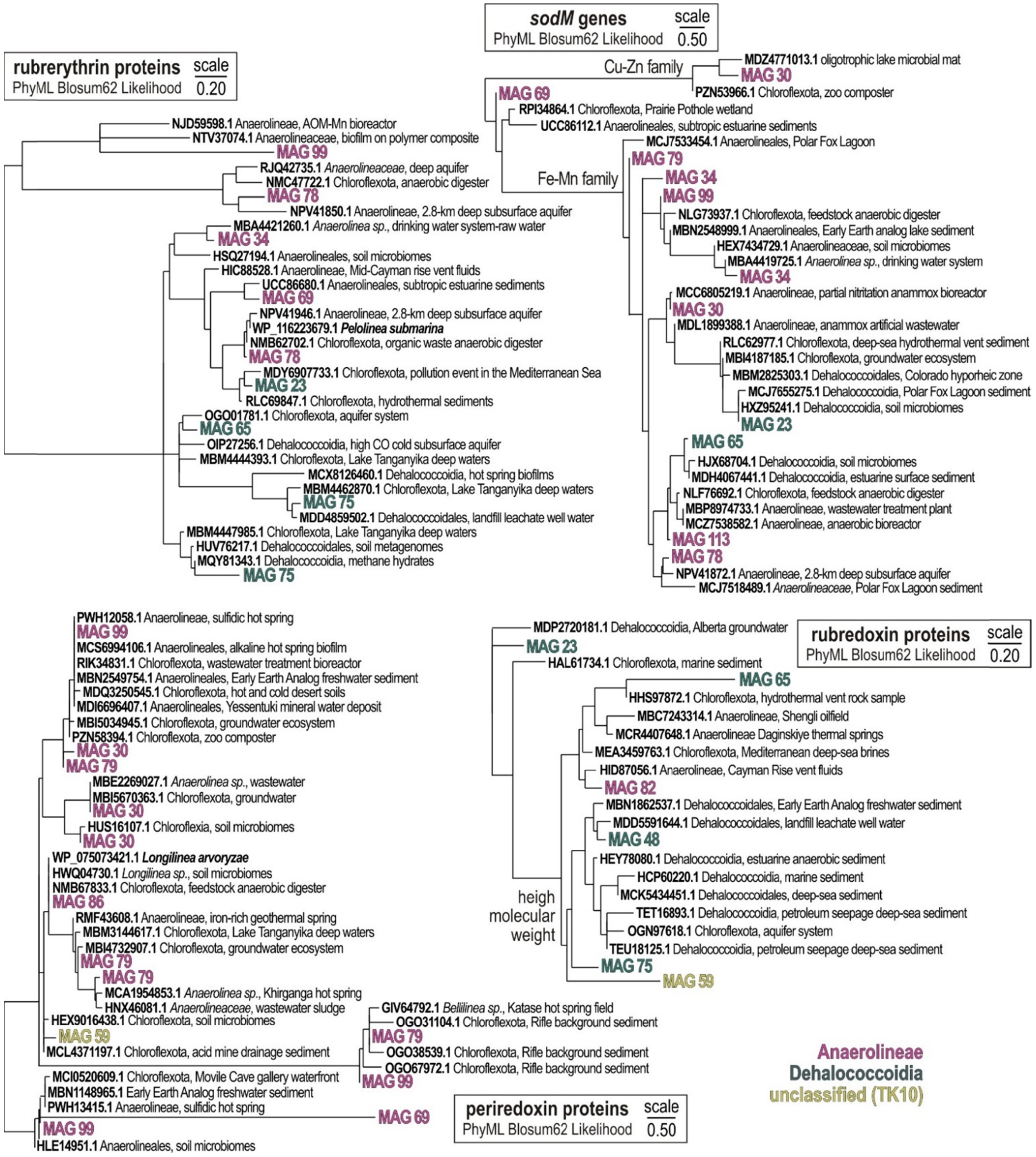

Supplementary Figure S4. Phylogenetic tree of marker genes related to antioxidant systems. PhyML Blosum62 phylogenetic tree of conserved regions for extracted ORFs encoding the superoxide dismutase (*sodM*, 19 amino acids), rubrerythrin (64 amino acids), rubredoxin (47 amino acids) and periredoxin (12 amino acids) proteins assigned to Chloroflexota based on 100 replicates. Boldface types signify cultivated species and NCBI sequence accession numbers.

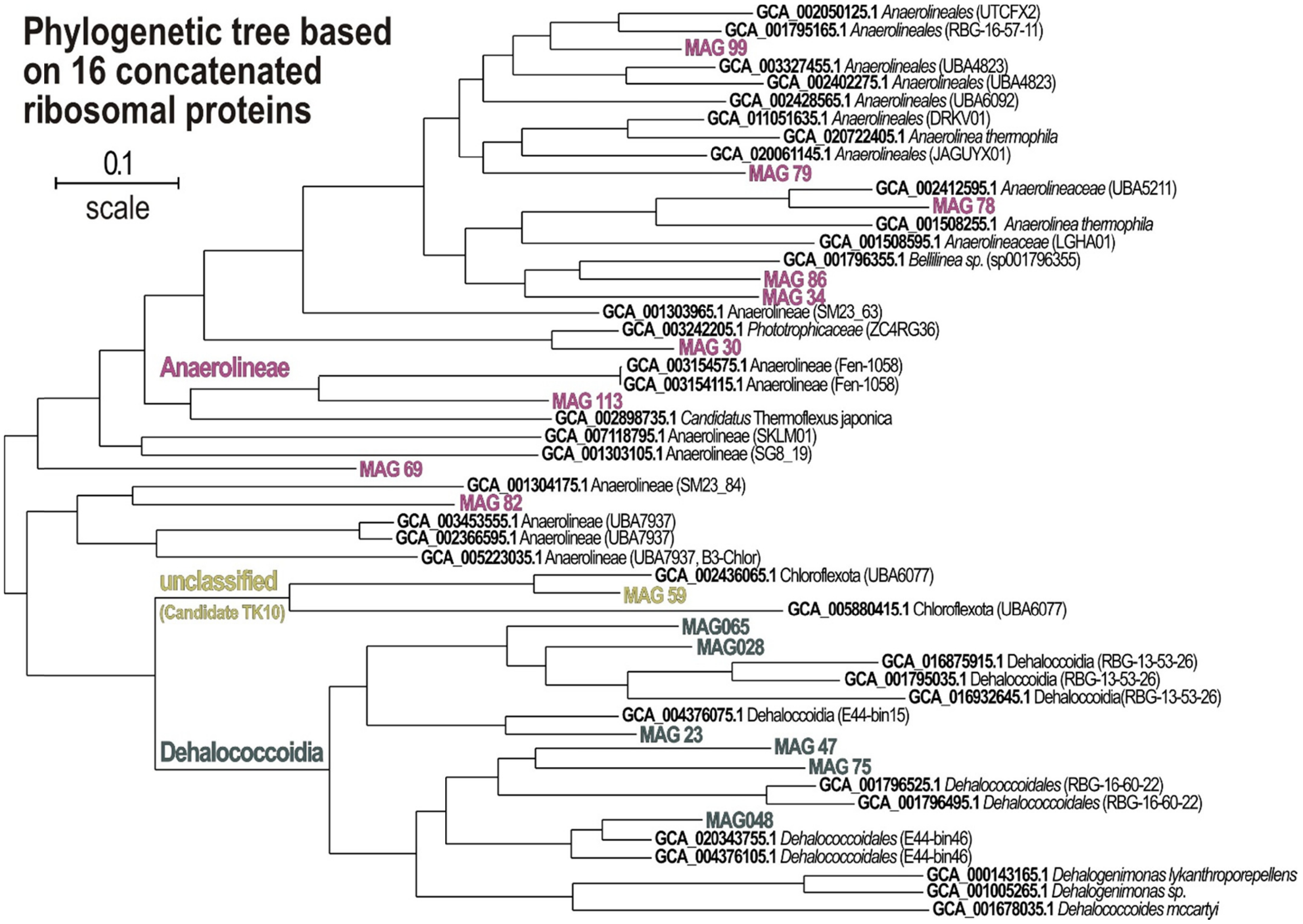

Supplementary Figure S5. Phylogenetic tree based on 16 concatenated ribosomal proteins extracted from all metagenome-assembled genomes assigned to Chloroflexota. Boldface types signify GTDB sequence accession numbers (Parks *et al*. 2022).

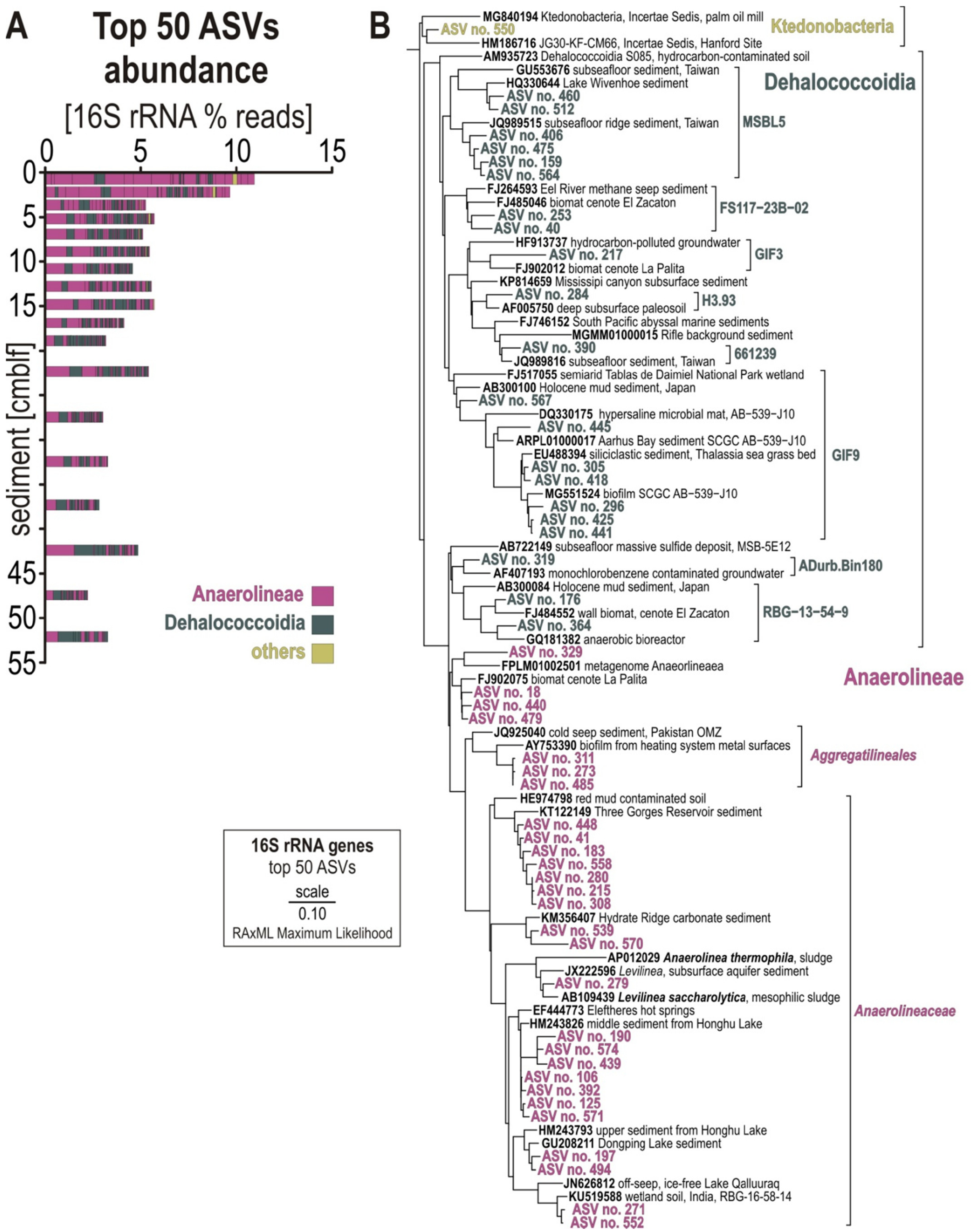

Supplementary Figure S6. (**A**) Relative abundances of the top 50 ASVs assigned to Chloroflexota with sediment depth; and (**B**) RaxML Maximum Likelihood phylogenetic tree of the corresponding partial 16S rRNA gene sequences (V4 hypervariable region).

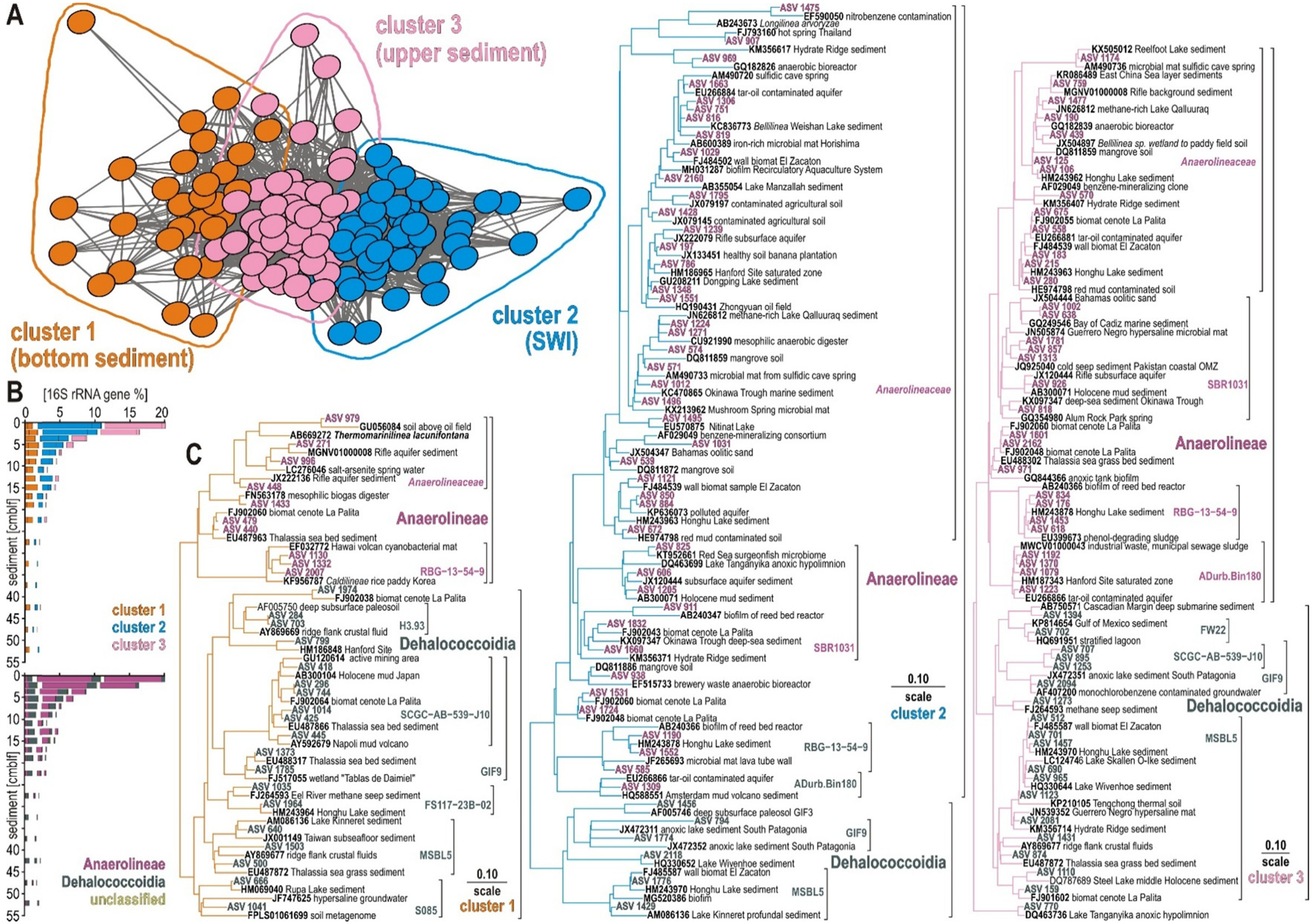

Supplementary Figure S6. (**A**) Network analysis of filtered amplicon variant sequences (128 ASVs) assigned to Chloroflexota; (**B**) bar charts of their corresponding abundances [16S rRNA gene %]; and (**C**) phylogenetic trees of 16S rRNA gene amplicons in cluster 1 (29 ASVs), cluster 2 (50 ASVs) and cluster 3 (49 ASVs). The three clusters can be interpreted in terms of bottom waters, sediment-water interface (SWI) and sediments. The main clades included in cluster 1, 2, and 3 are Dehalococcoidia GIF9, *Anaerolineaceae* and Dehaloccoidia MSBL5, respectively.

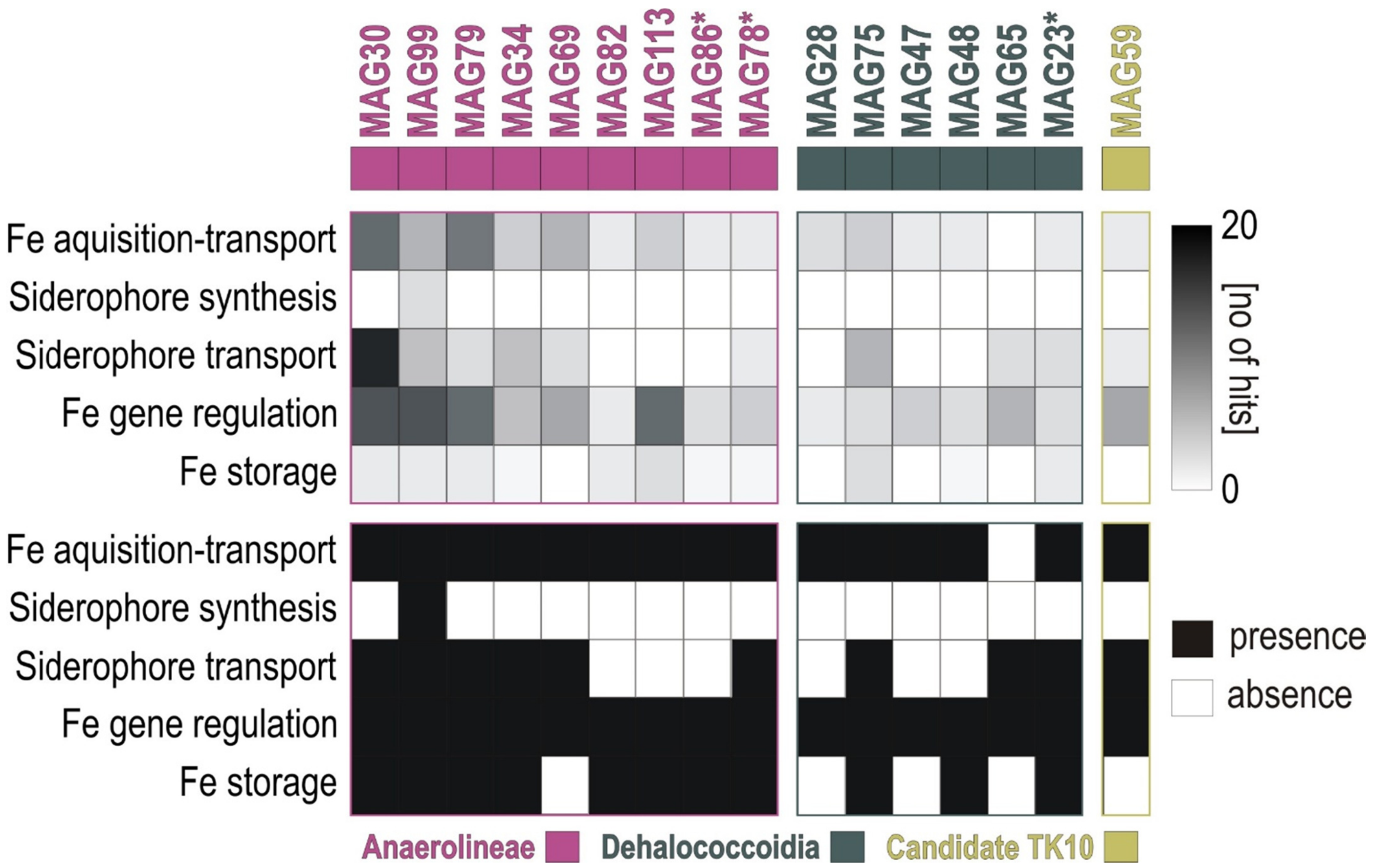

Supplementary Figure S7. Heatmaps of metabolic potential related to iron processes based on the FeGenie pipeline (Garber *et al*. 2020), according to the number of hits (**top**) and presence/absence (**bottom**) of the corresponding operons. Metabolic potential for dissimilatory iron reduction and/or oxidation was not identified.

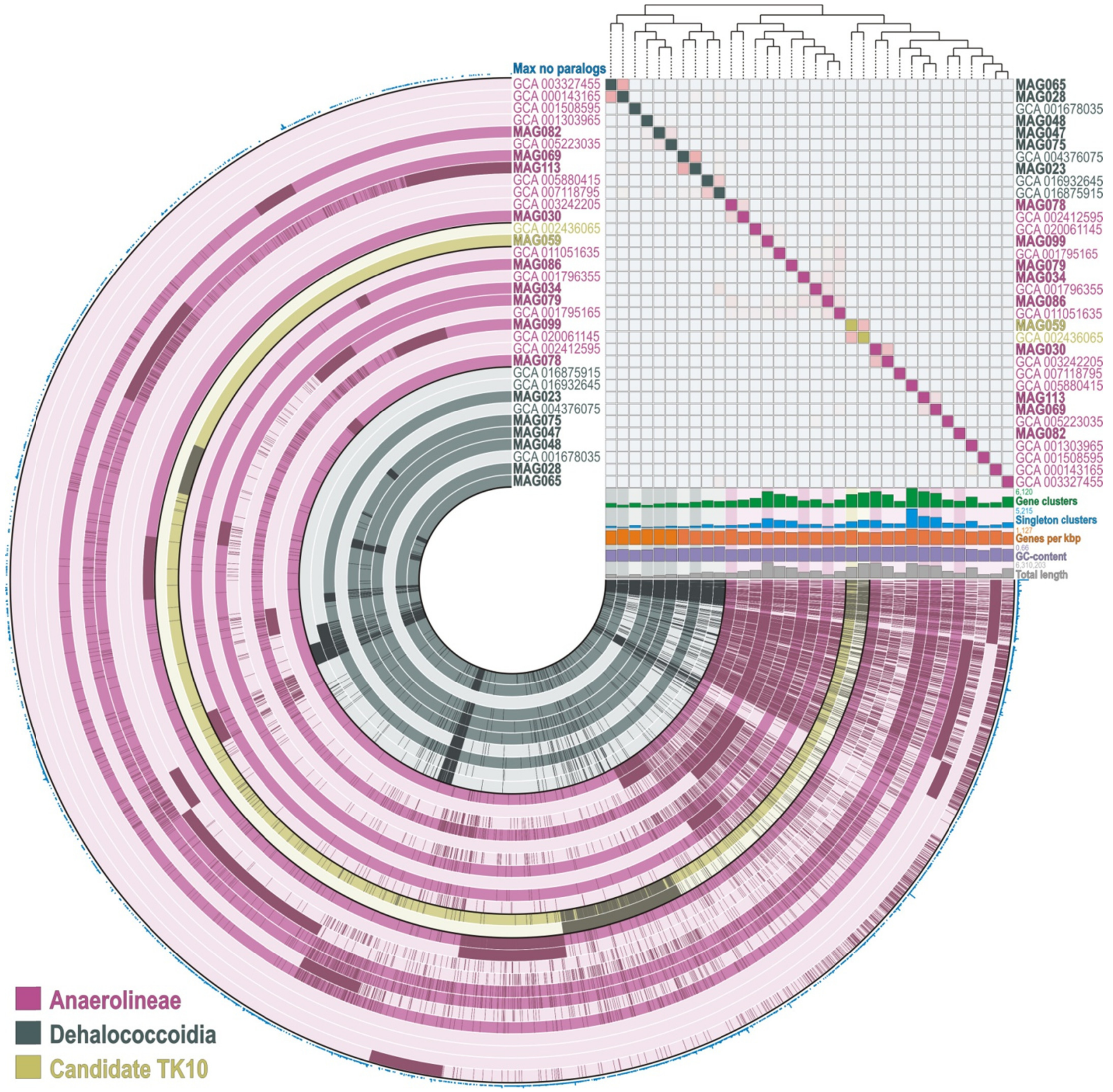

Supplementary Figure S8. Pangenome analysis including the 16 partial MAGs assigned to Chloroflexota in this study and 18 representative MAGs from the GTDB database (Parks *et al*. 2022). Alignments and data visualization were performed in anvi'o (Eren *et al*. 2015).

Supplementary Table S1. List of enzymes and gene abbreviations extracted from Open Reading Frames (ORFs).

Processes and pathways	Enzymes	Gene abbreviations
Nitrogen cycling	nitrogen fixation proteins	*nifS-U*
	ammonia-forming cytochrome c552 nitrite reductase	*nrfA*
	polysulphide reductase, *NrfD* family protein	*nrfD*
Sulfur cycling	dissimilatory sulfate reductase	*dsrA-G*
	anaerobic dimethyl-sulfoxide reductase	*dsmAB*
	assimilatory sulfite reductase	*sirA*
Arsenic cycling	arsenical pump-driving ATPase	*arsA*
	arsenite export protein	*arsB*
	arsenate reductase (LMWP family)	*arsC*
	arsenite S-adenosylmethyltransferase	*arsM*
	arsenical-resistance protein	*arsR*
Antioxidant systems	superoxide dismutase	*sodM*
	cytochrome c oxidase	*cycX*
	cytochrome bd oxidase	*cydX*
Hydrogen production	formate hydrogenlyase	*fhl*
	(non-)reducing F420 coenzyme hydrogenase	*mvhA*
	methyl-viologen hydrogenase	*mvhD*
	heterodisulfide reductase	*hdrA-F*
Hydrogen consumption	NAD-reducing hydrogenase	*hoxE*
	[NiFe] hydrogenase	*hypA-E*
Electron bifurcation	NADH-dependent flavin reductase	*noxA-B*
	NADH-ubiquinone oxidoreductase	*nuoA-N*
Refractory OM (β-oxidation)	haloacid dehalogenase	*HAD-II*
	benzoyl-CoA reductase	*bzdQ*
	3-hydroxyacyl-CoA dehydrogenase / 3-hydroxybutyryl-CoA dehydrogenase	*hbdAB*
	acyl-CoA dehydrogenase / butyryl-CoA dehydrogenase	*ACADs*
Glycolysis	sugar kinase, hexokinase	*HKN*
	glucose-6-phosphate isomerase	*GPI*
	1-phosphofructokinase	*PFK-1*
	fructose-2-phosphate aldolase	*ALDOA*
	triose-phosphate isomerase	*TPI*
	glyceraldehyde-tri-phosphate dehydrogenase	*GAPDH*
	phosphoglycerate kinase	*PGK*
	phosphoglycerate mutase	*PGM*
	enolase	*ENO1*
	pyruvate kinase	*PKM*
	acetyl-coenzyme A synthetase	*acs*
	aldehyde ferredoxin oxidoreductase	*ald-fdx*
Fermentation	pyruvate dehydrogenase	*pdh*
	phosphoenolpyruvate carboxylase	*PEPC*
	phosphoenolpyruvate carboxykinase	*PEPCK*
	pyruvate formate-lyase, formate C-acetyltransferase	*pfl*

Wood-Ljungdahl pathway (carbonyl-branch)	formate dehydrogenase	*fdh*
	formate-tetrahydrofolate synthetase/ligase	*fts*
	5,10-methylenetetrahydrofolate cyclohydrolase / dehydrogenase	*mthfc / mthfd*
	5,10-methylenetetrahydrofolate reductase	*mthfr*
	methyl-tetrahydrofolate: corrinoid methyltransferase	*mthmt*
	carbon monoxide dehydrogenase	*codh*
	acetyl-coenzyme A decarbonylase / synthase	*cdha*
	pyruvate ferredoxin oxidoreductase	*pyr-fdx*
	1,2,3-methylamine-specific coenzyme M methyltransferase (methyl-branch)	*mta, mtm, mtb, mtt*
	phosphotransacetylase	*pta*
	acetate kinase	*akn*
	butyrate kinase	*bkn*
	acyl-coenzyme A synthetase / ligase short chain (= acetate-CoA ligase)	*acss2*
TCA cycle	citrate synthase	*cs*
	aconitase, aconitate hydratase	*ACO*
	isocitrate dehydrogenase	*IDH*
	2-oxoglutarate synthetase	*2OGS*
	succinyl-coenzyme A synthetase (or ligase)	*SUCLG*
	succinate dehydrogenase	*SDH*
	fumarase, fumarate hydratase	*FH*
	malate dehydrogenase	*MDH*
	2-oxoglutarate ferredoxin oxidoreductase	*2-oxo-fdx*
RuBiSCO	ribulose-1,5-diphosphate carboxylase/oxygenase (form I large subunit)	*RuBisCo*
	acetyl-CoA carboxylase, biotin carboxylase	*ACC*
Cell energy storage (polyphosphate, glycogen, trehalose)	polyphosphate kinase	*ppk1*
	glycogen synthase	*gys*
	glycogen phosphorylase	*pyg*
	trehalose synthase	*treS*
	trehalose phosphatase	*tpp1*
	carbon storage	*crsA*
	gas vesicle protein	*gvp*
Taxonomy	RNA polymerase sigma factor	*RpoD*

Supplementary Table S2. Statistics of *de novo* co-assembly for Chloroflexota metagenome-assembled genomes.

MAG ref	Completeness	Contamination	Quality	Predicted genes	Identified ORFs	Taxonomy
MAG30	94.72	0.00	high	5310	1436	Chloroflexota Anaerolineae Aggregatilineales A4b ZC4RG36 (= *Phototrophicaceae*)
MAG99	94.55	8.97	good	5441	3382	Chloroflexota Anaerolineae Anaerolineales UBA4823 CTSoil-045
MAG79	93.64	4.73	high	3736	2056	Chloroflexota Anaerolineae Anaerolineales DRKV01
MAG34	91.82	3.64	high	2190	1418	Chloroflexota Anaerolineae Anaerolineales Anaerolineaceae PNON01
MAG69	77.73	2.12	good	4849	2540	Chloroflexota Anaerolineae 4572-78 NAK82
MAG82	76.82	4.77	good	2155	1176	Chloroflexota Anaerolineae UBA7937
MAG113	72.88	3.82	good	5461	2983	Chloroflexota Anaerolineae Thermoflexales Fen-1058
MAG86*	61.82	1.09	medium	1279	919	Chloroflexota Anaerolineae Anaerolineales Anaerolineaceae Longilinea
MAG78*	54.59	1.01	medium	2242	1329	Chloroflexota Anaerolineae Anaerolineales Anaerolineaceae Pelolinea
MAG28	98.18	2.00	high	2112	821	Chloroflexota Dehalococcoidia RBG-13-53-26 RBG-13-53-26
MAG75	78.05	5.94	good	1649	1332	Chloroflexota Dehalococcoidia Dehalococcoidales RBG-16-60-22
MAG47	69.85	0.22	good	1237	1101	Chloroflexota Dehalococcoidia Dehalococcoidales RBG-16-60-22 E44-bin89
MAG48	68.94	0.00	good	737	640	Chloroflexota Dehalococcoidia Dehalococcoidales E44-bin46 E44-bin46
MAG65	69.57	4.62	good	1514	1178	Chloroflexota Dehalococcoidia RBG-13-53-26 RBG-13-53-26
MAG23*	59.74	0.00	medium	1910	648	Chloroflexota Dehalococcoidia E44-bin15 E44-bin15 Kmv38
MAG59	80.91	3.96	good	4534	1946	Chloroflexota UBA6077 (= candidate TK10)

Supplementary Table S3. List of CAZyme families with enzyme names and targeted substrates. Entries were modified from (Zheng *et al.* 2023) according to the CAZy database (http://www.cazy.org/).

AA Family	Substrate high level	Auxiliary Activity Families - names, targeted substrates
AA01	lignin	laccase / p-diphenol:oxygen oxidoreductase / ferroxidase
AA01	lignin	laccase-like multicopper oxidase
AA02	lignin	cytochrome-c peroxidase
AA02	lignin	versatile peroxidase
AA02	lignin	lignin peroxidase
AA02	lignin	manganese peroxidase
AA02	lignin	ascorbate peroxidase
AA02	lignin	peroxidase
AA03	cellulose	cellobiose dehydrogenase
AA03	lignin	aryl alcohol oxidase
AA03	lignin	glucose 1-oxidase
AA03	lignin	alcohol oxidase
AA03	lignin	pyranose oxidase
AA04	lignin	vanillyl-alcohol oxidase
AA05	lignin	glyoxal oxidase
AA05	lignin	galactose oxidase
AA05	lignin	alcohol oxidase
AA05	lignin	oxidase with oxygen as acceptor
AA05	lignin	raffinose oxidase
AA06	lignin	1,4-benzoquinone reductase
AA07	cellooligosaccharide	cellooligosaccharide dehydrogenase
AA07	chitooligosaccharide	chitooligosaccharide oxidase
AA07	glucooligosaccharide	glucooligosaccharide oxidase
AA08	lignin	Iron reductase domain
AA09	cellulose	lytic cellulose monooxygenase (C4-dehydrogenating)
AA09	cellulose	lytic cellulose monooxygenase (C1-hydroxylating)
AA09	cellulose	copper-dependent lytic polysaccharide monooxygenases (LPMOs), cellulose
AA10	cellulose	lytic cellulose monooxygenase (C4-dehydrogenating; C1-hydroxylating)
AA10	chitin	lytic chitin monooxygenase
AA10	xylan	lytic xylan monooxygenase / xylan oxidase (glycosidic bond-cleaving)
AA11	chitin	copper-dependent lytic polysaccharide monooxygenases (LPMOs), chitin
AA12	polyphenol	pyrroloquinoline quinone-dependent oxidoreductase activity
AA13	starch	lytic starch monooxygenase / starch oxidase (glycosidic bond-cleaving)
AA14	xylan	copper-dependent lytic polysaccharide monooxygenases (LPMOs), xylan
AA14	xylan	lytic xylan monooxygenase / xylan oxidase (glycosidic bond-cleaving)
AA15	cellulose	lytic cellulose monooxygenase (C1-hydroxylating)
AA15	chitin	lytic chitin monooxygenase
AA16	cellulose	lytic cellulose monooxygenase (C1-hydroxylating)
AA17	pectin	lytic polygalacturonic acid monooxygenase (C4-hydroxylating)

CBM Family	Substrate high level	Carbohydrate-Binding Module Families - names, targeted substrates
CBM01	cellulose, chitin	ca. 40 residues almost exclusively in fungi, mediated by three aromatic residues
CBM02	cellulose, chitin, xylan	ca. 100 residues in a large number of bacterial enzymes, cellulose, chitin or xylan.

CBM03	cellulose, chitin	ca. 150 residues in bacterial enzymes, cellulose and chitin
CBM04	xylan, beta-glucan	ca. 150 residues in bacterial enzymes, xylan, beta-1,3-glucan, beta-1,3-1,4-glucan, beta-1,6-glucan, amorphous cellulose
CBM05	chitin	ca. 60 residues found in bacterial enzymes, chitin-binding, distantly related to the CBM12 family
CBM06	beta-glucan, xylan, cellulose	ca. 120 residues, amorphous cellulose, beta-1,4-xylan, beta-1,3-glucan, beta-1,3-1,4-glucan, and beta-1,4-glucan
CBM08	cellulose	cellulase from the slime mold *Dictyostelium discoideum*
CBM09	cellulose	ca. 170 residues in xylanases only
CBM10	cellulose	ca. 50 residues
CBM11	beta-glucan	ca. 180-200 residues, beta-1,4-glucan and beta-1,3-1,4-mixed linked glucans
CBM12	chitin	ca. 40-60 residues among chitinases binding chitin related to the CBM5 family
CBM13	xylan	ca. 150 residues of xylanase II in several plant lectins
CBM14	chitin	ca. 70 residues, chitin-binding function
CBM15	xylan	xylan- and xylooligosaccharide-binding
CBM16	cellulose, glucomannan	cellulose- and glucomannan-binding
CBM17	cellulose	ca. 200 residues, cellooligosaccharides, amorphous and derivatized cellulose
CBM18	chitin	ca. 40 residues, chitin-binding function, chitinase catalytic domains
CBM19	chitin	60-70 residues with chitin-binding function
CBM20	starch	granular starch-binding function with cyclodextrins, starch-binding domains (SBD)
CBM21	starch	ca. 100 residues, granular starch-binding function (SBD)
CBM22	xylan, beta-glucan	xylan-binding function, affinity with mixed beta-1,3/beta-1,4-glucans
CBM23	beta-mannan	mannan-binding function demonstrated in one case.
CBM24	beta-glucan	alpha-1,3-glucan (mutan)-binding function
CBM25	starch	starch-binding function
CBM26	starch	starch-binding function
CBM27	beta-mannan	mannan-binding function
CBM28	cellulose	endo-1,4-glucanase, binding non-crystalline cellulose, cellooligosaccharides, and beta-(1,3)(1,4)-glucans
CBM29	beta-mannan	binding to mannan/glucomannan
CBM30	cellulose	binding to cellulose
CBM31	xylan	binding to beta-1,3-xylan
CBM32	host glycan	binding to galactose, lactose, polygalacturonic acid, LacNAc (beta-D-galactosyl-1,4-beta-D-N-acetylglucosamine)
CBM33	cellulose, chitin	Copper-dependent lytic polysaccharide monooxygenases, now in family AA10
CBM34	starch	ca. 120 residues, granular starch-binding function
CBM35	xylan, beta-mannan, beta-galactan	ca. 130 residues, enzymes degrading xylan, soluble mannans, mannooligosaccharides, beta-galactan
CBM36	xylan	ca. 120-130 residues, calcium-dependent binding of xylans and xylooligosaccharides
CBM37	xylan, chitin, cellulose	ca. 100 residues, polysaccharide-degrading enzymes, specific to xylan, chitin, microcrystalline and swollen cellulose
CBM38	fructan	inulin-binding function
CBM39	beta-glucan,lipopolysaccharide	binding function with beta-1,3-glucan, lipopolysaccharide and lipoteichoic acid
CBM40	sialic acid	ca. 200 residues, sialic acid binding function
CBM41	beta-glucan	ca. 100 residues in bacterial pullulanases, alpha-glucans amylose, amylopectin, pullulan, oligosaccharide fragments
CBM42	xylan	ca. 160 residues binding to arabinofuranose
CBM43	beta-glucan	ca. 90-100 residues, beta-1,3-glucan binding function
CBM44	cellulose, xyloglucan	binding cellulose and xyloglucan
CBM45	starch	ca. 100 residues, alpha-amylases, alpha-glucan, water dikinases, starch-binding
CBM46	cellulose	ca. 100 residues, in several GH5 cellulases, cellulose-binding function
CBM47	fucose	ca 150 residues, fucose-binding activity

CBM48	glycogen	ca. 100 residues, glycogen-binding function and protein kinases
CBM49	cellulose	ca. 100 residues, in plant GH9 enzymes, binding crystalline cellulose
**CBM50**	chitin, peptidoglycan	ca. 50 residues, cleaving chitin, peptidoglycan, binding to chitopentaose, targeting petidoglycans
CBM51	host glycan	ca. 150 residues, binding to galactose
CBM52	beta-glucan	ca. 60 residues, binding to beta-1,3-glucan
CBM53	starch	starch-binding function
CBM54	xylan, beta-glucan, chitin	binding to xylan, yeast cell wall glucan, chitin
CBM55	chitin	binding to chitin
CBM56	beta-glucan	beta-1,3-glucan binding function
CBM57	glycosidases	domains attached to various glycosidases
CBM58	starch	binding to maltoheptaose
CBM59	mannan, xylan, cellulose	binding to mannan, xylan, and cellulose
CBM60	xylan	ca 120 residues in xylanases
CBM61	beta-galactan	ca. 150 residues in catalytic domains, beta-1,4-galactan binding function
CBM62	xyloglucan, arabinogalactan, galactomannan	binding galactose moieties, xyloglucan, arabinogalactan and galactomannan
CBM63	cellulose	binding to cellulose.
CBM64	cellulose	binding to cellulose
CBM65	xyloglucan	binding to a range of beta-glucans, preference for xyloglucan
CBM66	fructan	exo-acting beta-fructosidase, targets the terminal fructoside residue of fructans
CBM67	rhamnose	L-rhamnose binding activity
CBM68	starch	binding to maltotriose and maltotetraose
CBM69	starch	starch-binding function
CBM70	host glycan	hyaluronan-specific binding function
CBM71	host glycan	binding to lactose and LacNAc
CBM72	beta-glucan, xylan, and beta-mannan	130-180 residues, binding to polysaccharides, cellulose, beta-1,3/1,4-mixed linked glucans, xylan, beta-mannan
CBM73	chitin	ca 65 residues in enzymes active of chitin
CBM74	starch	ca. 300 residues in alpha-amylases, starch-binding function
CBM75	xyloglucan	290 residues with xyloglucan-binding function
CBM76	xyloglucan, glucomannan, beta-glucan	ca. 170 residues binding to beta-glucans, xyloglucan, glucomannan, beta-glucan
CBM77	pectin	ca. 110 residues, binding to pectins of low degree of esterification
CBM78	beta-glucan	ca. 150 residues, binding to decorated beta-1,4-glucans, preference for xyloglucan
CBM79	beta-glucan	ca. 130 residues, binding to various beta-glucans
CBM80	beta-glucan, beta-mannan	ca. 90 residues, specificity for beta-glycans (xyloglucan, glucomannan, galactomannan, barley beta-glucan)
CBM81	beta-glucan	ca. 100 residues in γ Proteobacteria, binding to beta-1,4-, beta-1,3,-glucans, xyloglucan, avicel, cellooligosaccharides
CBM82	starch	starch-binding function
CBM83	starch	starch-binding function
CBM84	xanthan	ca. 140 aminoacids in different families of CAZymes, xanthan-binding function
CBM85	cellulose, xylan, beta-glucan, beta-mannan	binding to glycans (cellulose, glucuronoxylan, b-1,3-1,4-glucan and glucomannan)
CBM86	xylan	binding to xylan
CBM87	galactosaminogalactan	binding to alpha-1,4-N-acetylgalactosamine-rich regions of galactosaminogalactan
CBM88	galactoxyloglucan and galactomannan	binding to terminal galactose in galactoxyloglucan and galactomannan
CBM89	xylan	binding to beechwood xylan and rye arabinoxylan
CBM90	ulvan	ca 110 residues in ulvan lyases, ulvan-binding function
CBM91	xylan	binding to xylans
CBM91	xylan	xylan binding activity, frequently associated with GH43
CBM92	carrageenan	carrageenan binding activity

CE Family	Substrate high level	Carbohydrate Esterase families - names, targeted substrates
**CE01**	polyphenol	cinnamoyl esterase
**CE01**	trehalose	trehalose 6-O-mycolyltransferase
**CE01**	xylan	feruloyl esterase
**CE01**	xylan	acetyl xylan esterase
**CE01**	xylan	carboxylesterase
**CE01**	xylan	diacylglycerol O-acyltransferase
CE02	xylan	acetyl xylan esterase
CE03	xylan	acetyl xylan esterase
CE04	chitin	chitin deacetylase
CE04	chitin	chitooligosaccharide deacetylase
CE04	peptidoglycan	peptidoglycan GlcNAc deacetylase
CE04	peptidoglycan	peptidoglycan N-acetylmuramic acid deacetylase
CE04	xylan	acetyl xylan esterase
CE05	cutin	cutinase
CE05	xylan	acetyl xylan esterase
CE06	xylan	acetyl xylan esterase
**CE07**	cephalosporin C	cephalosporin-C deacetylase
**CE07**	xylan	acetyl xylan esterase
CE08	pectin	pectin methylesterase
**CE09**	host glycan	N-acetylglucosamine 6-phosphate deacetylase
CE11	exo-polysaccharide	UDP-3-0-acyl N-acetylglucosamine deacetylase
CE12	pectin	pectin acetylesterase
CE12	pectin	rhamnogalacturonan acetylesterase
CE12	xylan	acetyl xylan esterase
CE13	pectin	pectin acetylesterase
**CE14**	chitin	chitin disaccharide deacetylase
**CE14**	chitin	diacetylchitobiose deacetylase
**CE14**	chitin	mycothiol S-conjugate amidase
**CE14**	exo-polysaccharide	N-acetyl-1-D-myo-inosityl-2-amino-2-deoxy-alpha-D-glucopyranoside deacetylase
CE15	lignin	4-O-methyl-glucuronoyl methylesterase
CE16	xylan	acetylesterase (active on various carbohydrate acetylesters)
CE17	beta-mannan	acetylmannan esterase
CE19	pectin	pectin methylesterase activity
CE20	xyloglucan	xyloglucan acetylesterase (XAC1771)

GH Family	Substrate_high_level	Glycoside Hydrolase families - names, targeted substrates
**GH001**	alkaloid	strictosidine beta-glucosidase
**GH001**	alkaloid	raucaffricine beta-glucosidase
**GH001**	beta-fucosides	beta-D-fucosidase
**GH001**	beta-galactan	beta-galactosidase
**GH001**	beta-galactan	6-phospho-beta-galactosidase
**GH001**	beta-glucan	beta-glycosidase
**GH001**	beta-glucan	beta-glucosidase
**GH001**	beta-glucan	exo-beta-1,4-glucanase
**GH001**	beta-glucan	6-phospho-beta-glucosidase
**GH001**	beta-glucuronan	beta-glucuronidase

**GH001**	beta-mannan	beta-mannosidase
**GH001**	human milk polysaccharide	lactase
**GH001**	polyphenol	beta-rutinosidase /alpha-L-rhamnose-(1,6)-beta-D-glucosidase
**GH001**	polyphenol	amygdalin beta-glucosidase
**GH001**	polyphenol	prunasin beta-glucosidase
**GH001**	polyphenol	vicianin hydrolase
**GH001**	polyphenol	thioglucosidase
**GH001**	polyphenol	beta-primeverosidase
**GH001**	polyphenol	isoflavonoid 7-O-beta-apiosyl-beta-glucosidase
**GH001**	polyphenol	ABA-specific beta-glucosidase
**GH001**	polyphenol	DIMBOA beta-glucosidase
**GH001**	polyphenol	protodioscin 26-O-Œ≤-D-glucosidase
**GH001**	polyphenol	phlorizin hydrolase
**GH001**	uric acid	hydroxyisourate hydrolase
**GH001**	xylan	beta-xylosidase
**GH002**	alpha-mannan	beta-D-galactofuranosidase
**GH002**	arabinan	alpha-L-arabinofuranosidase
**GH002**	beta-galactan	beta-galactosidase
**GH002**	beta-glucan	beta-glucosidase
**GH002**	beta-glucuronan	beta-glucuronidase
**GH002**	beta-mannan	beta-mannosidase
**GH002**	chitosan	exo-beta-glucosaminidase
**GH002**	host glycan	mannosylglycoprotein endo-beta-mannosidase
**GH002**	pectin	alpha-L-arabinopyranosidase
**GH002**	pectin	beta-galacturonidase
**GH002**	xylan	beta-xylosidase
**GH003**	arabinan	alpha-L-arabinofuranosidase
**GH003**	beta-glucan	exo-1,3-1,4-glucanase
**GH003**	beta-glucan	beta-1,2-glucosidase
**GH003**	beta-glucan	beta-1,3-glucosidase
**GH003**	beta-glucan	beta-glucosidase
**GH003**	beta-glucan	lichenase / endo-beta-1,3-1,4-glucanase
**GH003**	beta-glucan	glucan 1,4-beta-glucosidase
**GH003**	chitin	beta-N-acetylhexosaminidase
**GH003**	host glycan	beta-glucosylceramidase
**GH003**	peptidoglycan	beta-N-acetylglucosaminide phosphorylases
**GH003**	polyphenol	coniferin beta-glucosidase
**GH003**	polyphenol	protodioscin 26-O-Œ≤-D-glucosidase
**GH003**	xylan	xylan 1,4-beta-xylosidase
**GH003**	xyloglucan	isoprimeverose-producing oligoxyloglucan hydrolase
**GH003**	xyloglucan	xyloglucan-specific exo-beta-1,4-glucanase / exo-xyloglucanase
**GH004**	beta-glucan	6-phospho-beta-glucosidase
**GH004**	palatinose	palatinase
**GH004**	pectin	alpha-galacturonase
**GH004**	raffinose	alpha-galactosidase
**GH004**	starch	alpha-glucosidase
**GH004**	sucrose	maltose-6-phosphate glucosidase

**GH004**	xylan	alpha-glucuronidase
GH005	arabinan	alpha-L-arabinofuranosidase
GH005	arabinogalactan protein	beta-D-galactofuranosidase
GH005	arabinogalactan protein	endo-beta-1,6-galactanase
GH005	beta-glucan	endo-beta-1,3-glucanase / laminarinase
GH005	beta-glucan	endo-beta-1,4-glucanase / cellulase
GH005	beta-glucan	glucan beta-1,3-glucosidase
GH005	beta-glucan	lichenase / endo-beta-1,3-1,4-glucanase
GH005	beta-glucan	glucan endo-1,6-beta-glucosidase
GH005	beta-glycan	beta-glycosidase
GH005	beta-glycan	beta-primeverosidase
GH005	beta-mannan	mannan transglycosylase
GH005	beta-mannan	beta-1,3-mannanase
GH005	beta-mannan	glucomannan-specific endo-beta-1,4-glucanase
GH005	beta-mannan	beta-mannosidase
GH005	beta-mannan	mannan endo-beta-1,4-mannosidase
GH005	cellulose	exo-beta-1,4-glucanase / cellodextrinase
GH005	cellulose	cellulose beta-1,4-cellobiosidase
GH005	chitin	beta-N-acetylhexosaminidase
GH005	chitosan	chitosanase
GH005	glycolipid	steryl beta-glucosidase
GH005	glycolipid	endoglycoceramidase
GH005	glycolipid	beta-glucosylceramidase
GH005	glycolipid	beta-galactosylceramidase
GH005	polyphenol	beta-rutinosidase /alpha-L-rhamnose-(1,6)-beta-D-glucosidase
GH005	polyphenol	beta-glucosidase
GH005	xylan	arabinoxylan-specific endo-beta-1,4-xylanase
GH005	xylan	endo-beta-1,4-xylanase
GH005	xyloglucan	xyloglucan-specific endo-beta-1,4-glucanase
GH006	cellulose	endoglucanase
GH006	cellulose	lichenase / endo-beta-1,3-1,4-glucanase
GH006	cellulose	cellobiohydrolase
GH007	cellulose	reducing end-acting cellobiohydrolase
GH007	cellulose	endo-beta-1,4-glucanase
GH007	cellulose	endo-beta-1,3-1,4-glucanase
GH007	chitosan	chitosanase
GH008	beta-glucan	endo-beta-1,3(4)-glucanase / lichenase-laminarinase
GH008	beta-glucan	licheninase
GH008	cellulose	cellulase
GH008	chitosan	chitosanase
GH008	xylan	reducing-end-xylose releasing exo-oligoxylanase
GH008	xylan	endo-1,4-beta-xylanase
GH009	beta-glucan	endo-beta-1,4-glucanase (xanthanase)
GH009	beta-glucan	xyloglucan-specific endo-beta-1,4-glucanase / endo-xyloglucanase
GH009	beta-glucan	endo-beta-1,3(4)-glucanase / lichenase-laminarinase
GH009	beta-glucan	lichenase / endo-beta-1,3-1,4-glucanase
GH009	cellulose	endoglucanase

GH009	cellulose	exo-beta-1,4-glucanase / cellodextrinase
GH009	cellulose	cellobiohydrolase
GH009	chitosan	exo-beta-glucosaminidase
GH010	alkaloid	tomatinase
GH010	beta-glucan	endo-beta-1,4-glucanase
GH010	xylan	xylan endotransglycosylase
GH010	xylan	[retaining] arabinoxylan-specific endo-beta-1,4-xylanase
GH010	xylan	endo-1,3-beta-xylanase
GH010	xylan	endo-1,4-beta-xylanase
GH011	xylan	exo-1,4-beta-xylosidase
GH011	xylan	endo-beta-1,4-xylanase
GH012	beta-glucan	beta-1,3-1,4-glucanase
GH012	cellulose	endoglucanase
GH012	xyloglucan	xyloglucan endotransglycosylase
GH012	xyloglucan	xyloglucan hydrolase
**GH013**	alpha-glucan	oligosaccharide alpha-4-glucosyltransferase
**GH013**	alpha-glucan	branching enzyme
**GH013**	alpha-glucan	cyclomaltodextrin glucanotransferase
**GH013**	glucosylglycerate	Glucosylglycerate phosphorylase
**GH013**	glucosylglycerol	[retaining] glucosylglycerol phosphorylase
**GH013**	glycogen	4-alpha-glucanotransferase
**GH013**	starch	alpha-1,4-glucan: phosphate alpha-maltosyltransferase
**GH013**	starch	maltopentaose-forming alpha-amylase
**GH013**	starch	alpha-amylase
**GH013**	starch	[retaining] alpha-amylase
**GH013**	starch	oligo-alpha-glucosidase
**GH013**	starch	maltotriose-forming alpha-amylase
**GH013**	starch	maltogenic amylase
**GH013**	starch	neopullulanase
**GH013**	starch	malto-oligosyltrehalose trehalohydrolase
**GH013**	starch	alpha-glucosidase
**GH013**	starch	amylo-alpha-1,6-glucosidase
**GH013**	starch	pullulanase
**GH013**	starch	cyclomaltodextrinase
**GH013**	starch	maltotetraose-forming alpha-amylase
**GH013**	starch	isoamylase
**GH013**	starch	glucodextranase
**GH013**	starch	maltohexaose-forming alpha-amylase
**GH013**	starch	malto-oligosyltrehalose synthase
**GH013**	starch	trehalose synthase
**GH013**	sucrose	[retaining] sucrose 6(F)-phosphate phosphorylase
**GH013**	sucrose	amylosucrase
**GH013**	sucrose	sucrose phosphorylase
**GH013**	sucrose	[retaining] sucrose alpha-glucosidase
**GH013**	sucrose	isomaltulose synthase
**GH013**	sucrose	amino acid transporter
**GH013**	trehalose	trehalose-6-phosphate hydrolase

GH014	alpha-glucan	beta-amylase
GH015	starch	dextran dextrinase
GH015	starch	glucoamylase
GH015	starch	glucodextranase
GH015	trehalose	alpha,alpha-trehalase
GH016	agarose	beta-agarase
GH016	arabinogalactan protein	endo-beta-1,3-galactanase
GH016	beta-glucan	beta-transglycosidase
GH016	beta-glucan	endo-1,3-beta-glucanase / laminarinase
GH016	beta-glucan	endo-1,3(4)-beta-glucanase
GH016	beta-glucan	licheninase
GH016	carrageenan	beta-carrageenase
GH016	carrageenan	kappa-carrageenase
GH016	chitin	chitin beta-1,6-glucanosyltransferase
GH016	exo-polysaccharide	beta-glycosidase
GH016	host glycan	endo-beta-1,4-galactosidase
GH016	host glycan	keratan-sulfate endo-1,4-beta-galactosidase
GH016	host glycan	hyaluronidase
GH016	porphyran	[retaining] beta-porphyranase
GH016	xyloglucan	xyloglucan:xyloglucosyltransferase
GH016	xyloglucan	xyloglucanase
GH017	abscisic acid	ABA-specific beta-glucosidase
GH017	beta-glucan	beta-1,3-glucanosyltransglycosylase
GH017	beta-glucan	beta-1,3-glucosidase
GH017	beta-glucan	glucan endo-1,3-beta-glucosidase
GH017	beta-glucan	licheninase
GH018	chitin	Nod factor hydrolase
GH018	chitin	chitinase
GH018	host glycan	endo-beta-N-acetylglucosaminidase
GH018	peptidoglycan	peptidoglycan hydrolase with endo-beta-N-acetylglucosaminidase specificity
GH018	peptidoglycan	lysozyme
GH019	chitin	chitinase
GH019	chitin	chitinase
GH019	peptidoglycan	lysozyme
GH020	exo-polysaccharide	beta-1,6-N-acetylglucosaminidase
GH020	host glycan	beta-6-SO3-N-acetylglucosaminidase
GH020	host glycan	beta-hexosaminidase
GH020	human milk polysaccharide	lacto-N-biosidase
GH022	peptidoglycan	lysozyme type C
GH022	peptidoglycan	lysozyme type i
GH023	chitin	chitinase
GH023	peptidoglycan	lysozyme type G
GH023	peptidoglycan	peptidoglycan lyase (also known in the literature as peptidoglycan lytic transglycosylase)
GH024	peptidoglycan	lysozyme
GH025	peptidoglycan	lysozyme
GH026	beta-glucan	lichenase / endo-beta-1,3-1,4-glucanase
GH026	beta-mannan	mannobiose-producing exo-beta-mannanase

GH026	beta-mannan	exo-beta-1,4-mannobiohydrolase
GH026	beta-mannan	beta-mannanase
GH026	xylan	beta-1,3-xylanase
GH027	alpha-galactan	alpha-galactosidase
GH027	arabinogalactan protein	beta-L-arabinopyranosidase
GH027	host glycan	alpha-N-acetylgalactosaminidase
GH027	raffinose	galactan:galactan galactosyltransferase
GH027	starch	isomalto-dextranase
GH028	pectin	xylogalacturonan hydrolase
GH028	pectin	polygalacturonase
GH028	pectin	rhamnogalacturonase
GH028	pectin	rhamnogalacturonan alpha-1,2-galacturonohydrolase
GH028	pectin	exo-polygalacturonase
GH028	pectin	exo-polygalacturonosidase
GH029	host glycan	alpha-1,3/1,4-L-fucosidase
GH029	host glycan	alpha-L-fucosidase
GH029	host glycan	alpha-1,2-L-fucosidase
GH030	arabinogalactan protein	endo-beta-1,6-galactanase
GH030	beta-fucosides	beta-fucosidase
GH030	beta-glucan	beta-glucosidase
GH030	beta-glucan	beta-1,6-glucanase
GH030	beta-glucuronan	beta-glucuronidase
GH030	host glycan	glucosylceramidase
GH030	xylan	[reducing end] beta-xylosidase
GH030	xylan	glucuronoarabinoxylan endo-beta-1,4-xylanase
GH030	xylan	beta-xylosidase
GH030	xylan	endo-beta-1,4-xylanase
GH031	alpha-galactan	alpha-galactosidase
GH031	alpha-mannan	alpha-mannosidase
GH031	host glycan	alpha-N-acetylgalactosaminidase
GH031	polyphenol	sulfoquinovosidase
GH031	starch	oligosaccharide alpha-1,4-glucosyltransferase
GH031	starch	alpha-glucosidase
GH031	starch	alpha-1,3-glucosidase
GH031	starch	alpha-glucan lyase
GH031	sucrose	sucrase-isomaltase
GH031	xyloglucan	alpha-xylosidase
GH032	fructan	levan fructosyltransferase
GH032	fructan	[retaining] sucrose:sucrose 6-fructosyltransferase (6-SST)
GH032	fructan	cycloinulo-oligosaccharide fructanotransferase
GH032	fructan	sucrose:fructan 6-fructosyltransferase
GH032	fructan	fructan:fructan 1-fructosyltransferase
GH032	fructan	fructan:fructan 6G-fructosyltransferase
GH032	fructan	sucrose:sucrose 1-fructosyltransferase
GH032	fructan	fructan beta-(2,1)-fructosidase/1-exohydrolase
GH032	fructan	fructan beta-(2,6)-fructosidase/6-exohydrolase
GH032	fructan	invertase

GH032	fructan	beta-2,6-fructan 6-levanbiohydrolase
GH032	fructan	endo-levanase
GH032	fructan	endo-inulinase
GH032	fructan	exo-inulinase
GH033	pectin	Kdo hydrolase
GH033	sialic acid	trans-sialidase
GH033	sialic acid	sialidase or neuraminidase
GH033	sialic acid	anhydrosialidase
GH034	sialic acid	sialidase or neuraminidase
GH035	beta-galactan	beta-1,3-galactosidase
GH035	chitin	exo-beta-glucosaminidase
GH035	pectin	beta-galactosidase
GH036	alpha-galactan	alpha-galactosidase
GH036	host glycan	alpha-N-acetylgalactosaminidase
GH036	raffinose	stachyose synthase
GH036	raffinose	raffinose synthase
GH037	trehalose	alpha,alpha-trehalase
GH038	alpha-mannan	mannosyl-oligosaccharide alpha-1,3-mannosidase
GH038	host glycan	mannosyl-oligosaccharide alpha-1,2-mannosidase
GH038	host glycan	mannosyl-oligosaccharide alpha-1,3-1,6-mannosidase
GH038	host glycan	alpha-mannosidase
**GH039**	beta-galactan	beta-galactosidase
**GH039**	beta-glucan	beta-glucosidase
**GH039**	cellulose	exo-beta-1,4-glucanase / cellodextrinase
**GH039**	host glycan	alpha-L-iduronidase
**GH039**	xylan	beta-xylosidase
**GH039**	xylan	alpha-L-arabinofuranosidase
GH042	arabinan	alpha-L-arabinopyranosidase
GH042	arabinogalactan protein	beta-galactosidase
GH043	arabinan	alpha-1,2-L-arabinofuranosidase
GH043	arabinan	exo-alpha-1,5-L-arabinofuranosidase
GH043	arabinan	[inverting] exo-alpha-1,5-L-arabinanase
GH043	arabinan	[inverting] endo-alpha-1,5-L-arabinanase
GH043	arabinogalactan protein	exo-beta-1,3-galactanase
GH043	beta-galactan	beta-D-galactofuranosidase
GH043	xylan	beta-1,3-xylosidase
GH043	xylan	beta-xylosidase
GH043	xylan	alpha-L-arabinofuranosidase
GH043	xylan	xylanase
GH044	beta-glucan	endoglucanase
GH044	xyloglucan	xyloglucanase
GH045	beta-glucan	endoglucanase
GH045	beta-mannan	endo-beta-1,4-mannanase
GH045	xyloglucan	xyloglucan-specific endo-beta-1,4-glucanase / endo-xyloglucanase
GH046	chitosan	chitosanase
GH047	host glycan	alpha-mannosidase
GH048	cellulose	reducing end-acting cellobiohydrolase

GH048	cellulose	endo-beta-1,4-glucanase
GH048	chitin	chitinase
GH049	alpha-glucan	dextranase
GH049	alpha-glucan	isopullulanase
GH049	alpha-glucan	dextran 1,6-alpha-isomaltotriosidase
GH049	arabinan	sulfated arabinan endo-1,4-beta-L-arabinanase
GH050	agarose	beta-agarase
GH051	arabinan	beta-xylosidase
GH051	arabinan	alpha-L-arabinofuranosidase
GH051	beta-glucan	endoglucanase
GH051	xylan	endo-beta-1,4-xylanase
GH052	xylan	beta-xylosidase
GH053	arabinogalactan protein	endo-beta-1,4-galactanase
GH054	arabinan	alpha-L-arabinofuranosidase
GH054	xylan	beta-xylosidase
GH055	beta-glucan	laminarin-degrading enzyme
GH055	beta-glucan	endo-beta-1,3-glucanase
GH055	beta-glucan	exo-beta-1,3-glucanase
GH056	host glycan	chondroitin hydrolase
GH056	host glycan	hyaluronidase
GH057	alpha-galactan	alpha-galactosidase
GH057	alpha-glucan	amylopullulanase
GH057	alpha-glucan	cyclomaltodextrinase
GH057	beta-glucan	branching enzyme
GH057	starch	4-alpha-glucanotransferase
GH057	starch	alpha-amylase
GH058	sialic acid	endo-N-acetylneuraminidase or endo-sialidase
GH059	beta-galactan	beta-galactosidase
GH059	glycolipid	galactocerebrosidase
GH062	arabinan	alpha-L-arabinofuranosidase
GH063	alpha-glucan	mannosylglycerate alpha-mannosidase / mannosylglycerate hydrolase
GH063	alpha-glucan	alpha-glucosidase
GH063	alpha-glucan	glucosylglycerate hydrolase
GH063	alpha-glucan	alpha-1,3-glucosidase
GH063	host glycan	processing alpha-glucosidase
GH064	beta-glucan	beta-1,3-glucanase
GH065	alpha-glucan	2-O-alpha-glucopyranosylglycerol: phosphate beta-glucosyltransferase
GH065	alpha-glucan	trehalose-6-phosphate phosphorylase
GH065	alpha-glucan	kojibiose phosphorylase
GH065	alpha-glucan	nigerose phosphorylase
GH065	alpha-glucan	1,3-Œ±-oligoglucan phosphorylase
GH065	alpha-glucan	trehalose phosphorylase
GH065	alpha-glucan	maltose phosphorylase
GH065	alpha-glucan	alpha-glucosyl-1,2-beta-galactosyl-L-hydroxylysine alpha-glucosidase
GH065	alpha-glucan	alpha,alpha-trehalase
GH065	exo-polysaccharide	3-O-alpha-glucopyranosyl-L-rhamnose phosphorylase
GH066	alpha-glucan	cycloisomaltooligosaccharide glucanotransferase

GH066	alpha-glucan	dextranase
GH067	xylan	xylan alpha-1,2-glucuronidase
GH067	xylan	alpha-glucuronidase
GH068	fructan	levansucrase
GH068	fructan	inulosucrase
GH068	fructan	beta-fructofuranosidase
GH070	alpha-glucan	reuteransucrase
GH070	alpha-glucan	alpha-4,6-glucanotransferase
GH070	alpha-glucan	alpha-1,2-branched dextransucrase
GH070	alpha-glucan	alpha-4,3-glucanotransferase
GH070	alpha-glucan	alternansucrase
GH070	alpha-glucan	dextransucrase
GH071	alpha-glucan	alpha-1,3-glucanase
GH072	beta-glucan	beta-1,3-glucanosyltransglycosylase
GH073	peptidoglycan	peptidoglycan hydrolase with endo-beta-N-acetylglucosaminidase specificity
GH073	peptidoglycan	lysozyme
GH073	peptidoglycan	mannosyl-glycoprotein endo-beta-N-acetylglucosaminidase
GH074	cellulose	endoglucanase
GH074	xyloglucan	oligoxyloglucan reducing end-specific cellobiohydrolase
GH074	xyloglucan	xyloglucanase
GH075	chitosan	chitosanase
**GH076**	alpha-glucan	alpha-glucosidase
**GH076**	alpha-mannan	alpha-1,6-mannanase
**GH077**	starch	amylomaltase or 4-alpha-glucanotransferase
GH078	alpha-rhamnoside	alpha-L-rhamnosidase
GH078	pectin	rhamnogalacturonan alpha-L-rhamnohydrolase
GH079	arabinogalactan protein	beta-4-O-methyl-glucuronidase
GH079	arabinogalactan protein	beta-glucuronidase
GH079	host glycan	heparanase
GH079	host glycan	baicalin beta-glucuronidase
GH079	host glycan	hyaluronoglucuronidase
GH080	chitosan	chitosanase
GH081	beta-glucan	endo-beta-1,3-glucanase
GH082	carrageenan	Œπ-carrageenase
GH083	host glycan	neuraminidase
GH084	host glycan	[protein]-3-O-(GlcNAc)-L-Ser/Thr beta-N-acetylglucosaminidase
GH084	host glycan	hyaluronidase
GH084	host glycan	N-acetyl beta-glucosaminidase
GH085	host glycan	endo-beta-N-acetylglucosaminidase
GH086	agarose	beta-porphyranase
GH086	agarose	beta-agarase
GH087	alpha-glucan	alpha-1,3-glucanase
GH087	alpha-glucan	mycodextranase
GH088	host glycan	d-4,5-unsaturated beta-glucuronyl hydrolase
GH088	host glycan	unsaturated chondroitin disaccharide hydrolase
GH089	host glycan	alpha-N-acetylglucosaminidase
GH090	exo-polysaccharide	endorhamnosidase

GH091	fructan	difructofuranose 1,2':2,3' dianhydride hydrolase [DFA-IIIase]
GH091	fructan	inulin lyase [DFA-I-forming]
GH091	fructan	inulin lyase [DFA-III-forming]
GH092	host glycan	mannosyl-oligosaccharide alpha-1,3-mannosidase
GH092	host glycan	mannosyl-oligosaccharide alpha-1,6-mannosidase
GH092	host glycan	alpha-1,2-mannosidase
GH092	host glycan	alpha-1,3-mannosidase
GH092	host glycan	alpha-1,4-mannosidase
GH092	host glycan	mannosyl-1-phosphodiester alpha-1,P-mannosidase
GH092	host glycan	mannosyl-oligosaccharide alpha-1,2-mannosidase
GH092	host glycan	alpha-mannosidase
GH093	arabinan	exo-alpha-L-1,5-arabinanase
GH094	beta-glucan	Œ≤-1,2-oligoglucan phosphorylase
GH094	beta-glucan	laminaribiose phosphorylase
GH094	cellulose	cellobiose phosphorylase
GH094	cellulose	cellobionic acid phosphorylase
GH094	cellulose	cellodextrin phosphorylase
GH094	chitin	chitobiose phosphorylase
GH094	chitin	4-O-Œ≤-D-glucosyl-D-galactose phosphorylase
GH095	host glycan	alpha-L-fucosidase
GH095	pectin, xylan	alpha-L-galactosidase
GH095	xyloglucan, host glycan	alpha-1,2-L-fucosidase
GH096	agarose	alpha-agarase
GH097	alpha-glucan	alpha-glucosidase
GH097	alpha-glucan	alpha-galactosidase
GH097	alpha-glucan	glucoamylase
GH098	host glycan	blood-group endo-beta-1,4-galactosidase
GH098	xylan	endo-beta-1,4-xylanase
GH099	alpha-mannan	mannan endo-1,2-alpha-mannanase
GH099	host glycan	glycoprotein endo-alpha-1,2-mannosidase
GH100	sucrose	alkaline and neutral invertase
GH101	host glycan	endo-alpha-N-acetylgalactosaminidase
GH102	peptidoglycan	peptidoglycan lytic transglycosylase
GH103	peptidoglycan	peptidoglycan lytic transglycosylase
GH104	peptidoglycan	peptidoglycan lytic transglycosylase
GH105	pectin	d-4,5-unsaturated alpha-galacturonidase
GH105	pectin	unsaturated rhamnogalacturonyl hydrolase
GH105	ulvan	d-4,5-unsaturated beta-glucuronyl hydrolase
GH106	pectin	rhamnogalacturonan alpha-L-rhamnohydrolase
GH106	pectin	alpha-L-rhamnosidase
GH107	fucoidan	endo-alpha-1,4-L-fucanase
GH108	peptidoglycan	N-acetylmuramidase
GH109	host glycan	alpha-N-acetylgalactosaminidase
GH109	host glycan	beta-N-acetylhexosaminidase
GH110	host glycan	alpha-1,3-galactosidase
GH110	host glycan	alpha-galactosidase
GH111	host glycan	keratan sulfate hydrolase (endo-beta-N-acetylglucosaminidase)

GH112	human milk polysaccharide	lacto-N-biose phosphorylase or galacto-N-biose phosphorylase
GH112	human milk polysaccharide	D-galactosyl-beta-1,4-L-rhamnose phosphorylase
GH113	beta-mannan	beta-mannanase
GH114	alpha-mannan	endo-alpha-1,4-polygalactosaminidase
GH115	arabinogalactan protein	alpha-(4-O-methyl)-glucuronidase
GH115	xylan	xylan alpha-1,2-glucuronidase
GH116	exo-polysaccharide	beta-glucosidase
GH116	exo-polysaccharide	beta-xylosidase
GH116	exo-polysaccharide	beta-N-acetylglucosaminidase
GH116	host glycan	acid beta-glucosidase/beta-glucosylceramidase
GH117	agarose	beta-D-galactofuranosidase
GH117	agarose	alpha-neoagaro-oligosaccharide hydrolase
GH118	agarose	beta-agarase
GH119	starch	alpha-amylase
GH120	xylan	beta-xylosidase
GH121	arabinan	beta-L-arabinobiosidase
GH122	starch	alpha-glucosidase
GH123	host glycan	glycosphingolipid beta-N-acetylgalactosaminidase
GH123	host glycan	beta-N-acetylgalactosaminidase
GH124	cellulose	endoglucanase
GH125	alpha-mannan	exo-alpha-1,6-mannosidase
GH126	alpha-glucan	alpha-amylase
GH127	arabinogalactan protein	beta-L-arabinofuranosidase
GH127	carrageenan	alpha-1,3-(3,6)-anhydro-D-galactosidase
GH127	pectin	3-C-carboxy-5-deoxy-L-xylose (aceric acid) hydrolase
GH128	beta-glucan	beta-1,3-glucosidase
GH128	beta-glucan	beta-1,3-glucanase
GH129	carrageenan	alpha-1,3-(3,6)-anhydro-D-galactosidase
GH129	host glycan	alpha-N-acetylgalactosaminidase
GH130	beta-mannan	beta-1,2-oligomannan phosphorylase
GH130	beta-mannan	beta-1,2-mannobiose phosphorylase
GH130	beta-mannan	beta-1,4-mannosylglucose phosphorylase
GH130	beta-mannan	beta-1,4-mannooligosaccharide phosphorylase
GH130	beta-mannan	Œ≤-1,2-oligomannan phosphorylase
GH130	beta-mannan	Œ≤-1,2-mannobiose phosphorylase
GH130	beta-mannan	beta-1,4-mannosyl-N-acetyl-glucosamine phosphorylase
GH130	beta-mannan	beta-1,2-mannosidase
GH131	beta-glucan	broad specificity exo-beta-1,3/1,6-glucanase with endo-beta-1,4-glucanase activity
GH132	beta-glucan	beta-1,3-glucosidase
GH133	glycogen	amylo-alpha-1,6-glucosidase
GH134	beta-mannan	endo-beta-1,4-mannanase
GH135	alpha-galactan	alpha-1,4-galactosaminogalactan hydrolase
GH136	human milk polysaccharide	Lacto-N-biosidase (Lewis antigen a/b specificity)
GH136	human milk polysaccharide	lacto-N-biosidase
GH137	pectin	beta-L-arabinofuranosidase
GH138	pectin	rhamnogalacturonan alpha-1,2-galacturonohydrolase
GH139	pectin	alpha-2-O-Me-L-fucosidase

GH140	pectin	beta-1,2-apiosidase
GH141	pectin	alpha-L-fucosidase
GH141	xylan	xylanase
GH142	pectin	beta-L-arabinofuranosidase
GH143	pectin	2-keto-3-deoxy-D-lyxo-heptulosaric acid hydrolase
GH144	beta-glucan	beta-1,2-glucooligosaccharide sophorohydrolase
GH144	beta-glucan	endo-beta-1,2-glucanase
GH146	arabinan	beta-L-arabinofuranosidase
GH147	beta-galactan	beta-galactosidase
GH147	beta-galactan	beta-galactosidase
GH148	beta-glucan	beta-1,3-glucanase
GH148	beta-mannan	beta-1,3-glucanase
GH149	beta-glucan	beta-1,3-glucan phosphorylase
GH150	carrageenan	Iota-carrageenase
GH151	human milk polysaccharide	alpha-L-fucosidase
GH152	beta-glucan	beta-1,3-glucanase
GH153	exo-polysaccharide	poly-beta-1,6-D-glucosamine hydrolase
GH154	arabinogalactan protein	beta-1,6-D-glucuronidase
GH154	arabinogalactan protein	beta-glucuronidase
GH156	host glycan	exo-alpha-sialidase
GH157	beta-glucan	endo-beta-1,3-glucanase / laminarinase
GH158	beta-glucan	endo-beta-1,3-glucanase
GH159	beta-galactan	beta-D-galactofuranosidase
GH160	exo-polysaccharide	endo-beta-1,4-galactosidase
GH161	beta-glucan	beta-1,3-glucan phosphorylase
GH162	beta-glucan	endo-beta-1,2-glucanase
GH163	host glycan	endo-beta-N-acetylglucosaminidase cleaving GlcNAc-beta-1,2-Man
GH164	beta-mannan	beta-mannosidase
GH165	beta-galactan	beta-galactosidase
GH166	alpha-galactan	alpha-1,4-galactosaminogalactan hydrolase
GH167	carrageenan	beta-carrageenase
GH168	fucoidan	endo-alpha-(1,3)-L-fucanase
GH169	exo-polysaccharide	beta-1,4-D-glucuronidase
GH169	exo-polysaccharide	beta-glucuronidase
GH170	peptidoglycan	6-phospho-N-acetylmuramidase
GH171	peptidoglycan	peptidoglycan beta-N-acetylmuramidase
GH172	fructan	difructose-anhydride synthase
GH173	pectin	Œ≤-galactosidase (EC 3.2.1.23)
GH174	starch, glyocogen	fucoidan (Ib) endo-α-1,3-L-fucosidase (EC 3.2.1.-)
GH175	beta-glucan	beta-glucosidase (EC 3.2.1.21), wide specificity for beta-D-glucosides
GH176	amylopectin	isoamylase (EC 3.2.1.68)
**GH177**	host glycan	exo-α-sialidase (EC 3.2.1.18)
**GH178**	mannan	mannan endo-α-1,4-mannosidase (EC 3.2.1.-)
**GH179**	chitin	beta-N-acetylhexosaminidase (EC 3.2.1.52)

GT Family	Substrate high level	Glycosyl Transferases families - names, targeted substrates
For detailed information on GT substrates, please go to: http://www.cazy.org/GlycosylTransferases		

**GT002**	large branched glycan polymers	various glucosyltransferases, glycogen phosphorylase (EC 2.4.1.-)
**GT004**	large branched glycan polymers	various glucosyltransferases, glycogen phosphorylase (EC 2.4.1.-)
**GT028**	diacylglycerols with long-chain acyl groups	1,2-diacylglycerol 3-glucosyltransferase (EC 2.4.1.157), generates glycolipids as surrogates for phospholipids under phosphate deprivation
**GT035**	large branched glycan polymers	alpha-1,4-glucan phosphorylase (EC 2.4.1.1)
**GT041**	substrate proteins	peptide N-β-glucosyltransferase (EC 2.4.1.-)
**GT041**	substrate proteins	protein O-α-L-fucosyltransferase (EC 2.4.1.221)
**GT041**	substrate proteins	protein O-β-N-acetylglucosaminyltransferase (EC 2.4.1.255)
**GT83**	L-arabinose	undecaprenyl phosphate-α-L-Ara4N: 4-amino-4-deoxy-β-L-arabinosyltransferase

**PL Family**	**Substrate high level**	**Polysaccharide Lyase families - names, targeted substrates**
PL01	pectin	pectin lyase
PL01	pectin	pectate lyase
PL01	pectin	exo-pectate lyase
PL02	pectin	pectate lyase
PL02	pectin	exo-polygalacturonate lyase
PL03	pectin	pectate lyase
PL04	pectin	rhamnogalacturonan endolyase
PL05	alginate	alginate lyase
PL05	host glycan	endo-beta-1,4-glucuronan lyase
PL06	alginate	poly(alpha-L-guluronate) lyase / G-specific alginate lyase
PL06	alginate	oligoalginate lyase / exo-alginate lyase
PL06	alginate	MG-specific alginate lyase
PL06	alginate	alginate lyase
PL06	host glycan	chondroitinase B
PL07	alginate	alpha-L-guluronate lyase / G-specific alginate lyase
PL07	alginate	oligoalginate lyase / exo-alginate lyase
PL07	alginate	poly-(MG)-lyase / MG-specific alginate lyase
PL07	alginate	poly(beta-mannuronate) lyase / M-specific alginate lyase
PL07	host glycan	endo-beta-1,4-glucuronan lyase
PL08	alginate	poly(beta-mannuronate) lyase / M-specific alginate lyase
PL08	host glycan	hyaluronate lyase
PL08	host glycan	chondroitin ABC lyase
PL08	host glycan	chondroitin AC lyase
PL08	host glycan	heparin lyase / heparin lyase I
PL08	xanthan	xanthan lyase
PL09	pectin	pectate lyase
PL09	pectin	rhamnogalacturonan endolyase
PL09	pectin	exopolygalacturonate lyase
PL10	pectin	pectate lyase
PL11	pectin	rhamnogalacturonan endolyase
PL11	pectin	rhamnogalacturonan exolyase
PL12	host glycan	heparin lyase / heparin lyase I
PL12	host glycan	heparin-sulfate lyase
PL13	host glycan	heparin lyase
PL14	alginate	exo-oligoalginate lyase
PL14	alginate	poly(beta-mannuronate) lyase / M-specific alginate lyase
PL14	host glycan	beta-1,4-glucuronan lyase

PL15	alginate	oligoalginate lyase / exo-alginate lyase
PL15	alginate	alginate lyase
PL15	host glycan	heparin lyase / heparin lyase I
PL15	host glycan	heparin-sulfate lyase / heparin lyase III
PL16	host glycan	hyaluronan lyase
PL17	alginate	oligoalginate lyase
PL17	alginate	alginate lyase
PL18	alginate	poly(alpha-L-guluronate) lyase / G-specific alginate lyase
PL18	alginate	alginate lyase
PL20	host glycan	endo-beta-1,4-glucuronan lyase
PL21	host glycan	heparin lyase
PL21	host glycan	heparin-sulfate lyase
**PL22**	pectin	oligogalacturonate lyase / oligogalacturonide lyase
PL26	pectin	rhamnogalacturonan exolyase
PL29	host glycan	chondroitin-sulfate ABC endolyase
PL30	host glycan	hyaluronate lyase
PL31	alginate	poly(beta-mannuronate) lyase / M-specific alginate lyase
PL31	host glycan	endo-beta-1,4-glucuronan lyase
PL32	alginate	poly(beta-mannuronate) lyase / M-specific alginate lyase
PL33	gellan	gellan lyase
PL33	host glycan	hyaluronate lyase
PL33	host glycan	chondroitin sulfate lyase
PL36	alginate	poly(beta-mannuronate) lyase / M-specific alginate lyase
PL37	host glycan	chondroitin-sulfate ABC endolyase
PL37	host glycan	heparin-sulfate lyase / heparin lyase III
PL38	host glycan	endo-beta-1,4-glucuronan lyase
PL41	alginate	alginate lyase
PL42	arabinogalactan protein	L-rhamnose-Œ±-1,4-D-glucuronate lyase
PL42	arabinogalactan protein	L-RhŒ±-Œ±-1,4-GlcA Œ±-L-rhamnohydrolase
